# Evaluating the effect of *Clostridium difficile* conditioned medium on fecal microbiota community structure

**DOI:** 10.1038/s41598-017-15434-1

**Published:** 2017-11-27

**Authors:** Sabina Horvat, Aleksander Mahnic, Martin Breskvar, Saso Dzeroski, Maja Rupnik

**Affiliations:** 1 University of Maribor, Faculty of Medicine, Maribor, Slovenia; 2 National Laboratory for Health, Environment and Food, Maribor, Slovenia; 3 Jozef Stefan Institute, Ljubljana, Slovenia; 4 Jozef Stefan International Postgraduate School, Ljubljana, Slovenia; 5 Centre of Excellence for Integrated Approaches in Chemistry and Biology of Proteins, Ljubljana, Slovenia

## Abstract

*Clostridium difficile* infection (CDI) is typically associated with disturbed gut microbiota and changes related to decreased colonization resistance against *C. difficile* are well described. However, nothing is known about possible effects of *C. difficile* on gut microbiota restoration during or after CDI. In this study, we have mimicked such a situation by using *C. difficile* conditioned medium of six different *C. difficile* strains belonging to PCR ribotypes 027 and 014/020 for cultivation of fecal microbiota. A marked decrease of microbial diversity was observed in conditioned medium of both tested ribotypes. The majority of differences occurred within the phylum *Firmicutes*, with a general decrease of gut commensals with putative protective functions (i.e. *Lactobacillus*, *Clostridium_XIVa*) and an increase in opportunistic pathogens (i.e. *Enterococcus*). Bacterial populations in conditioned medium differed between the two *C. difficile* ribotypes, 027 and 014/020 and are likely associated with nutrient availability. Fecal microbiota cultivated in medium conditioned by *E. coli*, *Salmonella* Enteritidis or *Staphylococcus epidermidis* grouped together and was clearly different from microbiota cultivated in *C. difficile* conditioned medium suggesting that *C. difficile* effects are specific. Our results show that the changes observed in microbiota of CDI patients are partially directly influenced by *C. difficile*.

## Introduction

*Clostridium difficile* is recognized as the main cause of infectious diarrhoea associated with hospitalization and is also becoming an important cause of intestinal infections in the community^1,2^. Crucial for the development of *C. difficile* infection (CDI) is the disturbance of the normal gut microbiota, which is usually due to treatment with antibiotics^1,2^. Several *in vivo* and *in vitro* models have been used to study interactions between gut microbiota and *C. difficile* ^3,4^. In addition, different patient populations have been studied in respect to changes of gut microbiota associated with *C. difficile* colonization^5,6^. Individuals colonized with *C. difficile* have less diverse gut microbiota, with the general decrease of anaerobic bacteria (e.g. *Bacteroides, Bifidobacterium*) and an increased proportion of facultative anaerobes (e.g. *Enterobacteriaceae, Enterococcus*)^5,6^. Not only changes within particular bacterial groups, but also specific microbial patterns are associated with *C. difficile* colonization^7^. However, recent findings suggest that CDI development is not associated with specific gut microbial composition, but rather with the presence of a limited set of bacterial species and their functional capacities^8–12^.

Gut microbiota could interact with *C. difficile* through several mechanisms^13,14^, including nutrient competition, regulation of sporulation and vegetative growth and modulation of immune response. The best studied mechanism by which gut commensal bacteria (e.g. *Clostridium scindens*) confer colonization resistance is modification of host-derived bile salts^12,15–17^. Depletion of bile salts metabolites (i.e. secondary bile acid deoxycholate), due to absent bacterial groups in disturbed microbiota, can permit *C. difficile* growth^12^. Specimens from CDI patients contain significantly lower levels of secondary bile acids compared to samples from healthy controls^18^. On the other hand, the reduction of several bacterial taxa after antibiotic treatment leads to an increased proportion of primary bile acid taurocholate that favours *C. difficile* germination^19,20^. Another possible mechanism of colonization resistance is through production of the short chain fatty acid (SCFA), butyrate. Butyrate plays a central role in maintaining gut homeostasis^21^. Butyrate-producing bacteria are found within the *Lachnospiraceae* and *Ruminococcaceae* families; taxa that are greatly reduced in stool specimens from CDI patients^22^. In nutrient competition, sialic acid is one of the important factors. Gut commensal bacteria (e.g. *Bacteroides thetaiotaomicron*) release sialic acids from mucus. Several bacterial groups that would normally metabolize free sialic acid are not present anymore upon antibiotic treatment and this allows *C. difficile* to use this nutrient source and expand its population in the gut^23^. The gut symbiont *B. thetaiotaomicron* also produces high levels of succinate, another key nutrient promoting *C. difficile* expansion after antibiotic treatment^24^. *C. difficile* growth is also favoured in the abundance of some other carbon sources (i.e. mannitol, sorbitol) in altered gut microbiota^25^. Furthermore, the development of CDI is favoured because microbiota changes are associated with unbalanced immune responses after antibiotic treatment^26^.

While mechanisms through which gut microbiota influences *C. difficile* are being partially elucidated, not much is known about the possible effect of *C. difficile* on gut microbiota. During CDI, *C. difficile* colonizes the intestine with dysbiotic microbiota and could represent a significant proportion of the bacterial population. In a mouse model, *C. difficile* was shown to be associated with other species in mucus-associated bacterial communities^27^. Moreover, in a gnotobiotic mouse model with simplified 12 species murine microbiota (Oligo-MM^12^), colonization with *C. difficile* resulted in significant compositional perturbation^28^. Another recent study demonstrated that epidemic *C. difficile* isolates successfully compete for nutrients even in the presence of a complex gut microbiota^29^.

It is very likely that *C. difficile* overgrowth in the gut niche affects the reconstitution of gut microbiota during and after the CDI. To address the possible impact of *C. difficile* on the growth of common gut bacteria, we analyzed the differences in bacterial community structure of fecal microbiota after *in vitro* growth in *C. difficile* conditioned medium. Representative strains from two PCR ribotypes were used. PCR ribotype 027 was selected because it was previously associated with more disturbed microbiota than others^7,30,31^, while PCR ribotype 014/020 was selected as the most commonly isolated ribotype on global scale^32^. To characterize the specific changes in the structure of the fecal microbiota community cultivated in *C. difficile* conditioned medium, several data analysis approaches were used, including the LEfSe algorithm (in the mothur software) and three different machine learning methods. LEfSe uses linear discriminant analysis (LDA) to find operational taxonomic units (OTUs) that differ significantly in abundance between samples^33^. The machine learning method ReliefF (WEKA software) ranks the OTUs in such a way, that the highest ranking OTUs would best differentiate between the ribotypes (and controls)^34^. The machine learning methods jRip (WEKA software) and predictive clustering trees (PCT, CLUS software) provide interpretable models (rules and trees), that can be used to predict target values (such as ribotype and relative OTU abundances) and explain the predictions through the model structure^35–37^.

## Results

### *C. difficile* conditioned medium decreases the diversity of fecal microbiota *in vitro*

Two growth media were tested and showed major differences supporting the growth of diverse microbial communities. The stability of the community composition grown in Wilkins Chalgren Anaerobe Broth (WCAB) was better as compared to that grown in Anaerobe Basal Broth (ABB) after a 5 day incubation period (Fig. S1, Table S1). In experiments with the ABB medium, significant changes were observed already between controls and original uncultivated pooled fecal samples (Fig. S1). Hence, this medium was not included in further analyses.

**Figure 1:**
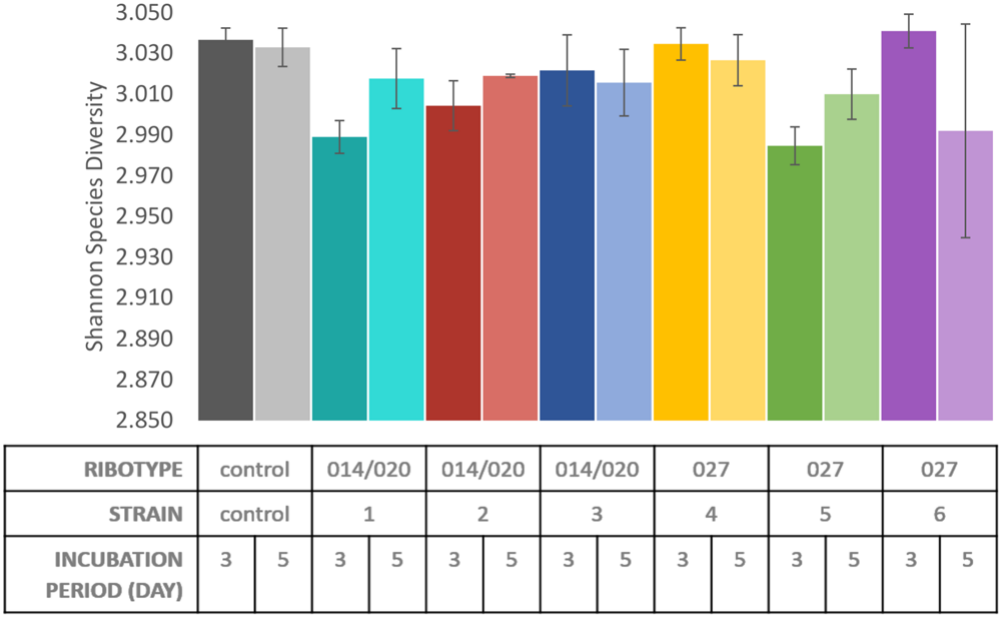
Species diversity (Shannon index with corresponding standard deviation) in WCAB (Wilkins Chalgren Anaerobe Broth) control medium and in WCAB conditioned medium of six different *C. difficile* strains (1–6), belonging to two different ribotypes (014/020, 027) on day 3 and day 5.

When comparing the growth between unconditioned control WCAB growth medium and *C. difficile* conditioned WCAB growth medium, an effect on fecal microbiota diversity was detected. We compared the diversity for each day (3 and 5) separately and for each strain separately, to the diversity of the unconditioned control growth medium. The Shannon diversity index values of communities grown in conditioned medium as compared to those grown in control medium were significantly lower for three out of six tested strains (t-test: p = 0.003–0.047) only on day 3 and not on day 5 (Fig. 1). The decrease of the Shannon diversity index in conditioned medium was not ribotype associated, as decreased diversity was observed with strains that belonged to ribotype 027 (1 strain) and ribotype 014/020 (2 strains).

### *C. difficile* conditioned medium affects specific groups within fecal microbiota and changes are ribotype associated

**Figure 2:**
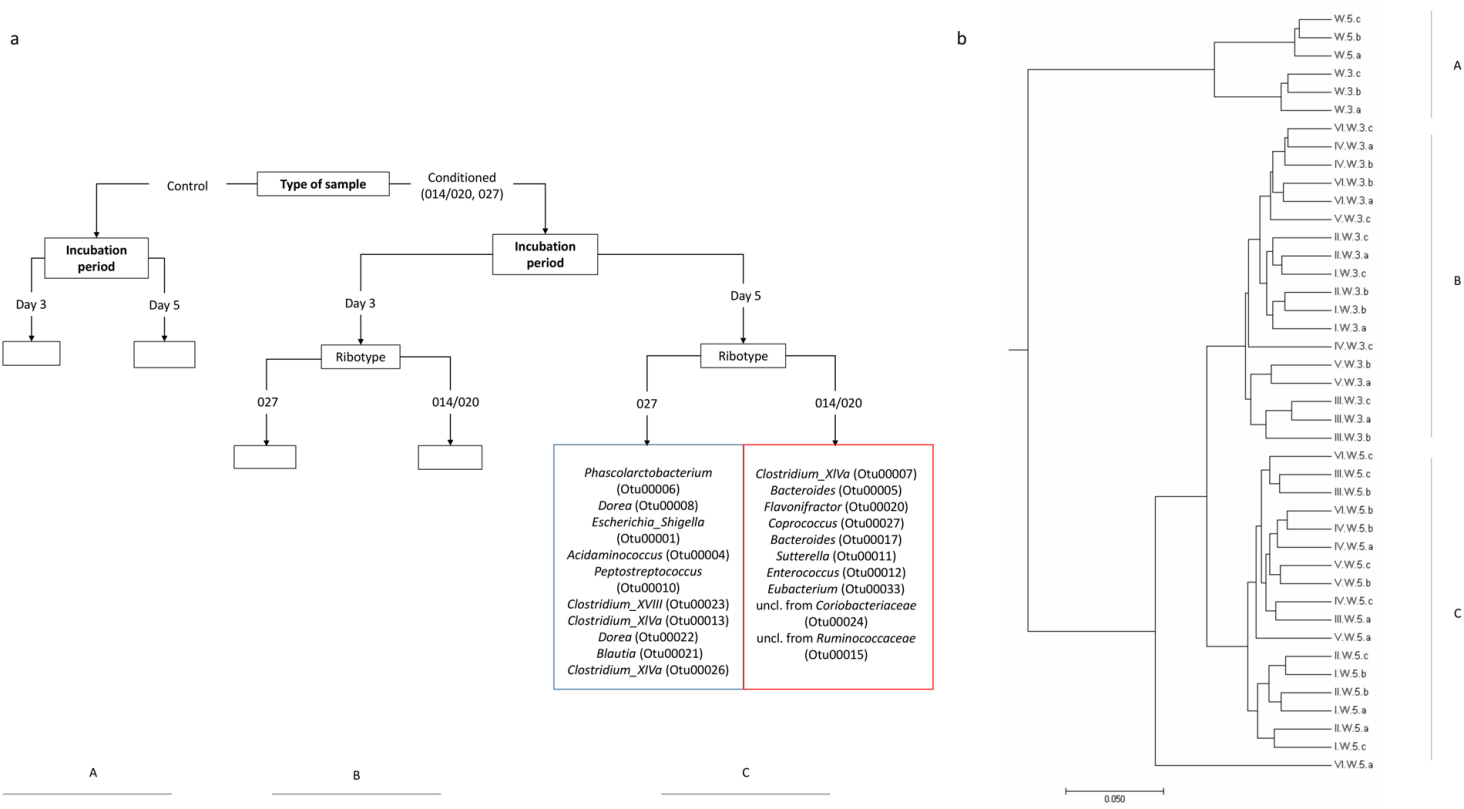
Clustering of samples according to (**a**) machine learning (PCT, CLUS software) and (**b**) mothur analysis (tree viewed by MEGA software, v.7.0.26). Samples clustered in three groups, i.e. group of WCAB (Wilkins Chalgren Anaerobe Broth) control medium samples (segment A), group of WCAB conditioned medium samples after three days of incubation (segment B) and group of WCAB conditioned medium samples after five days of incubation (segment C). A predictive clustering tree (PCT) predicts the OTU composition of a sample from the environmental conditions and the ribotype. The leaves of the tree, corresponding to clusters of samples with similar OTU composition, predict the relative abundances of the OTUs, while the path to each leaf from the root of the tree explains the cluster in terms of the environmental conditions. During PCT construction, tests for the internal nodes of the tree are chosen to maximize the similarity in OTU composition for the samples that go to the same branch and maximize the differences between the samples that go to the left and the right branch, respectively (segments A, B, C). The segment C shows the difference in OTU composition between the fifth and the sixth leaf node from the left. The fifth node represents OTUs associated with ribotype 027 (blue box), the sixth node represents OTUs associated with ribotype 014/020 (red box), after five day incubation period in WCAB growth medium. The differences in values of relative abundances between the two ribotypes for OTUs are presented in Fig. 4b.

**Figure 3:**
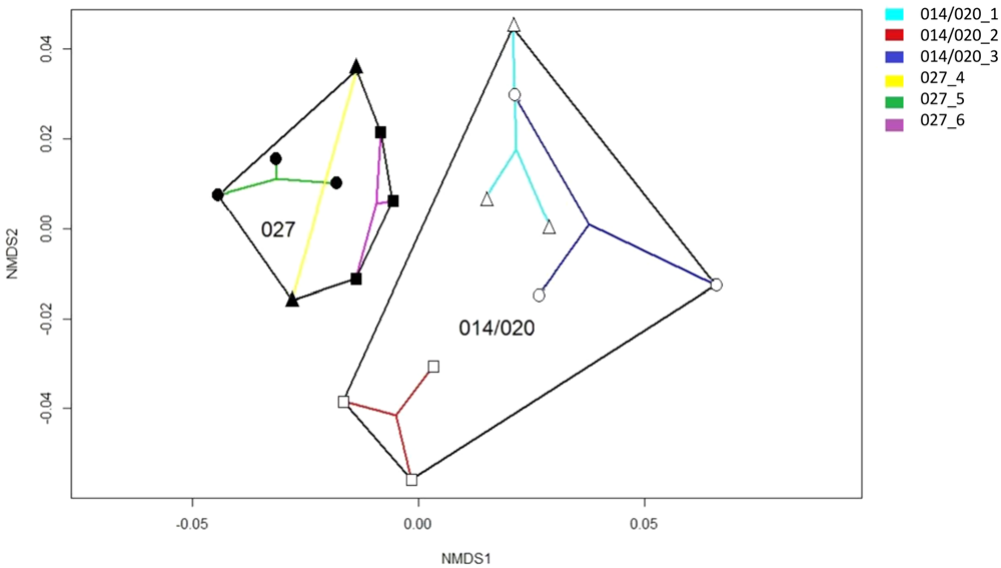
Non-Metric Multidimensional Scaling (NMDS) analysis for samples cultured in WCAB (Wilkins Chalgren Anaerobe Broth) after a five day incubation period (R, vegan package). Black lines separate two *C. difficile* ribotypes, whereas colored lines indicate different *C. difficile* strains.

In the first step all samples were grouped either by hierarchical clustering or by learning a PCT (Fig. 2). Both methods distributed our samples and ranked the growth conditions in the same way; the conditioned medium had the highest impact on the grouping, followed by day of incubation and finally by ribotype (014/020 vs. 027). Significant differentiation between ribotypes was noticed with NMDS analysis (R, vegan package; AMOVA: p < 0.001) and within the PCT only after 5 days of incubation (Fig. 2a, panel C; Fig. 3). Differences among the three strains within each ribotype were not statistically significant.

**Figure 4:**
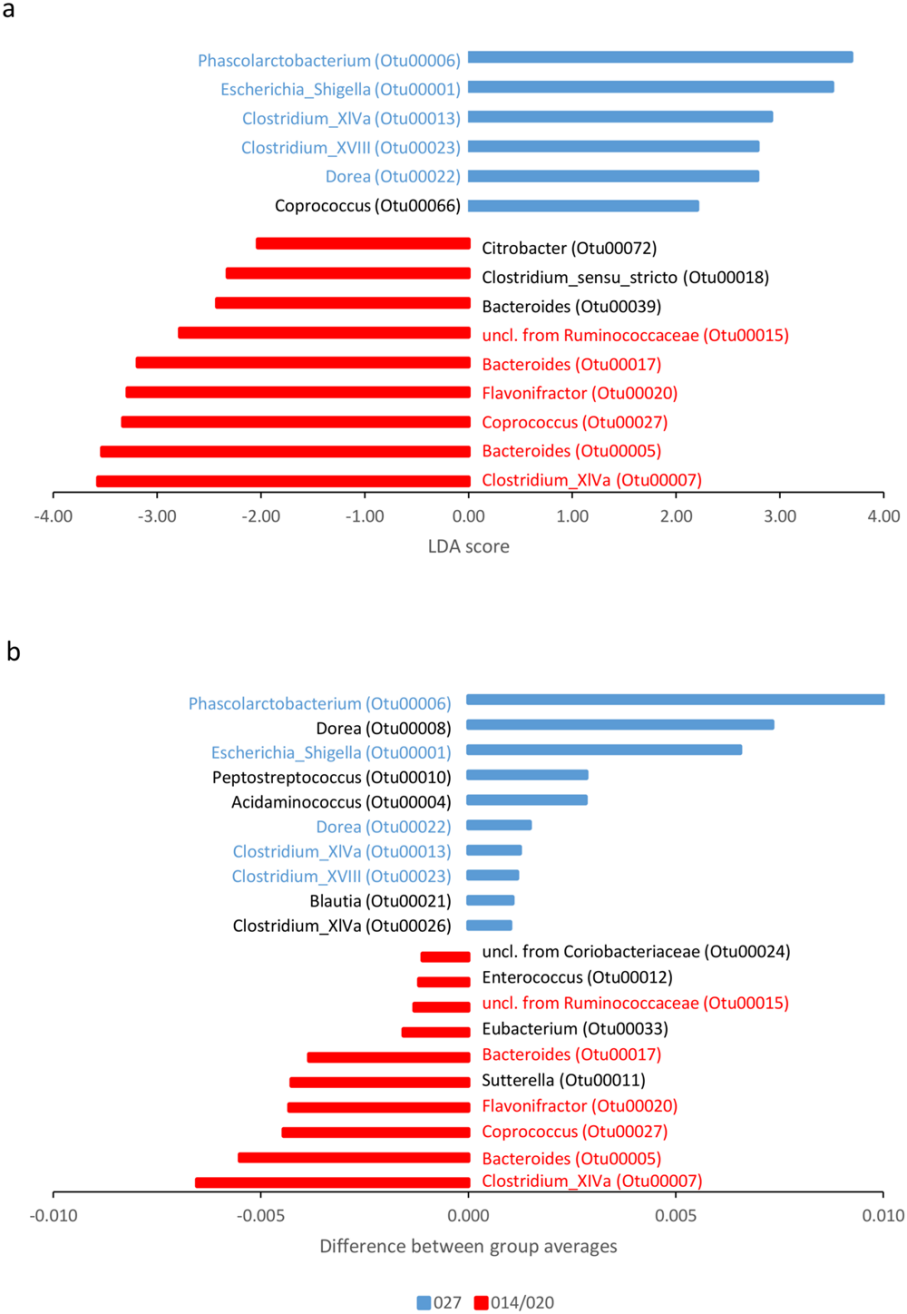
OTUs associated with WCAB (Wilkins Chalgren Anaerobe Broth) conditioned medium of ribotype 027 () or ribotype 014/020 (), on day 5, selected by using (**a**) the mothur software (LEfSe test) and (**b**) machine learning (PCT, CLUS software). Blue font marks OTUs increased in ribotype 027 conditioned medium, red font marks OTUs increased in ribotype 014/020 conditioned medium, detected with both data analysis approaches, respectively. For the mothur analysis, only the LDA scores of significant OTUs are shown. For the machine learning analysis, the values represent the difference of OTU relative abundances between the left and right branch of the segment C in the PCT (shown in Fig. 2).

Changes in OTUs were analysed with LEfSe and the machine learning algorithms and are presented for different pairs of sample types (014/020 and control; 027 and control; 014/020 and 027) (Tables 1 and S2; Figs. 2, 4 and S2).

**Table 1:** Differentially represented OTUs identified by the LEfSe test (mothur software) and machine learning approaches (WEKA ReliefF).

analysis	LEfSe test		LEfSe test		Machine learning
status	increased		decreased		Ranked OTUs (WEKA ReliefF)
day	3	5	3	5	
014/020 in comparison to control (Comparison 2)	*Bacteroides* (Otu00002)	*Bacteroides* (Otu00002)	*Escherichia_Shigella* (Otu00001)	*Escherichia_Shigella* (Otu00001)	*Clostridium_sensu_stricto* (Otu00018)*Bacteroides (Otu00025)*Escherichia_Shigella* (Otu00001)*Bacteroides (Otu00005)*Bacteroides* (Otu00002)uncl. *Burkholderiales* (Otu00045)*Streptococcus* (Otu00096)*Enterococcus* (Otu00012)*Erysipelotrichaceae_incertae_sedis* (Otu00083)*Coprococcus* (Otu00027)*Lactobacillus* (Otu00059)*Streptococcus* (Otu00019)*Odoribacter* (Otu00089)uncl. *Ruminococcaceae* (Otu00065)*Dorea* (Otu00022)*Bacteroides* (Otu00041)*Flavonifractor* (Otu00020)*Bacteroides* (Otu00076)uncl. *Ruminococcaceae* (Otu00015)*Clostridium_XlVa* (Otu00007)
	*Bacteroides* (Otu00005)	*Dorea* (Otu00008)	*Clostridium_sensu_stricto* (Otu00018)	*Veillonella* (Otu00003)	
	*Clostridium_XlVa* (Otu00007)	*Bacteroides* (Otu00005)	*Veillonella* (Otu00003)	*Clostridium_sensu_stricto* (Otu00018)	
	*Dorea* (Otu00008)	*Clostridium_XlVa* (Otu00007)	*Streptococcus* (Otu00019)	*Streptococcus* (Otu00019)	
	*Enterococcus* (Otu00012)	uncl. *Lachnospiraceae* (Otu00014)	*Dorea* (Otu00022)	*Dorea* (Otu00022)	
	*Bacteroides* (Otu00017)	*Enterococcus* (Otu00012)	*Bacteroides* (Otu00009)	*Bacteroides* (Otu00009)	
	*Coprococcus* (Otu00027)	*Coprococcus* (Otu00027)	*Clostridium_XIVa* (Otu00013)	*Blautia* (Otu00021)	
	*Sutterella* (Otu00011)	*Sutterella* (Otu00011)	*Lactobacillus* (Otu00059)	*Oscillibacter* (Otu00038)	
	*Flavonifractor* (Otu00020)	*Flavonifractor* (Otu00020)	*Alistipes* (Otu00035)	*Clostridium_XIVa* (Otu00013)	
	*Phascolarctobacterium* (Otu00006)	*Parabacteroides* (Otu00016)	uncl. *Ruminococcaceae* (Otu00065)	*Lactobacillus* (Otu00059)	
027 in comparison to control (Comparison 3)	*Bacteroides* (Otu00002)	*Bacteroides* (Otu00002)	*Escherichia_Shigella* (Otu00001)	*Escherichia_Shigella* (Otu00001)	*Clostridium_sensu_stricto* (Otu00018)*Escherichia_Shigella (Otu00001)*Bacteroides (Otu00025)*Bacteroides* (Otu00005)*Streptococcus* (Otu00096)*Bacteroides* (Otu00002)*Lactobacillus* (Otu00059)uncl. *Ruminococcaceae* (Otu00065)*Coprococcus* (Otu00027)*Erysipelotrichaceae_incertae_sedis* (Otu00083)*Citrobacter* (Otu00072)uncl. *Ruminococcaceae* (Otu00015)*Parabacteroides* (Otu00016)*Enterococcus* (Otu00012)*Bacteroides* (Otu00041)*Clostridium_XlVa* (Otu00007)uncl. *Ruminococcaceae (*Otu00034)*Faecalibacterium* (Otu00032)
	*Bacteroides* Otu00005)	*Dorea* (Otu00008)	*Clostridium_sensu_stricto* (Otu00018)	*Veillonella* (Otu00003)	
	*Dorea* (Otu00008)	uncl. *Lachnospiraceae* (Otu00014)	*Veillonella* (Otu00003)	*Clostridium_sensu_stricto* (Otu00018)	
	*Clostridium_XlVa* (Otu00007)	*Clostridium_XlVa* (Otu00007)	*Streptococcus* (Otu00019)	*Streptococcus* (Otu00019)	
	*Bacteroides* (Otu00017)	*Bacteroides* (Otu00005)	*Dorea* (Otu00022)	*Dorea* (Otu00022)	
	*Enterococcus* (Otu00012)	*Enterococcus* (Otu00012)	*Bacteroides* (Otu00009)	*Bacteroides* (Otu00009)	
	*Peptostreptococcus* (Otu00010)	*Coprococcus* (Otu00027)	*Lactobacillus* (Otu00059)	*Oscillibacter* (Otu00038)	
	*Coprococcus* (Otu00027)	*Parabacteroides* (Otu00016)	*Clostridium_XIVa* (Otu00013)	*Blautia* (Otu00021)	
	*Phascolarctobacterium* (Otu00006)	*Bacteroides* (Otu00025)	*Alistipes* (Otu00035)	*Lactobacillus* (Otu00059)	
	*Sutterella* (Otu00011)	uncl *Ruminococcaceae* (Otu00034)	uncl. *Ruminococcaceae* Otu00065)	*Alistipes* (Otu00035)	

Uncl. = unclassified. LEfSe uses linear discriminant analysis (LDA) to find OTUs that significantly differ in abundance between control and *C. difficile* samples. Only top 10 OTUs with highest LDA scores are presented, for additional information see Table S2 in Supplementary Information. Additional information can be extracted from the WEKA ReliefF rankings of OTUs in terms of their relevance for distinguishing between control and different ribotype *C. difficile* samples. Top 20 ranked OTUs are shown in the rightmost column (taken from Figure S2).

When using LEfSe algorithm, OTUs that significantly decreased in *C. difficile* conditioned medium according to the control were *Escherichia_Shigella*, *Clostridium_sensu_stricto*, *Veillonella*, *Streptococcus*, *Dorea*, *Bacteroides*, *Lactobacillus* and an unclassified member of *Ruminococcaceae* (Table 1) on day 3. On day 5, both ribotypes were also associated with a decrease in *Blautia* (Table 1). The same genera, but different OTUs (e.g. *Dorea, Bacteroides*) could be also increased in conditioned medium (Table 1). Bacterial groups with increased proportions in conditioned medium of both ribotypes included *Enterococcus* and an unclassified member of *Burkholderiales* (Table 1). Additionally, ribotype 014/020 was associated with an increase in *Sutterella*.

Among the machine learning approaches, we first used PCTs and learned to predict OTU composition from the incubation period and type of sample (control, ribotype 014/020 and ribotype 027). OTUs that contribute most to the differentiation between microbial communities in conditioned medium on day 5 are given in Fig. 2a (panel C). The top 10 OTUs, ranked in terms of their difference in relative abundance between the two ribotypes are shown.

For the second machine learning task, we used the relative OTU abundances to predict the type of the sample (conditioned medium of specific ribotype). For this purpose, we used the RIPPER algorithm for learning classification rules, i.e. its implementation jRip in the WEKA software. The OTUs identified by jRip as relevant for distinguishing between control and *C. difficile* conditioned medium samples were *Escherichia_Shigella*, *Phascolarctobacterium* and *Clostridium_sensu_stricto* (Fig. S2).

Rather than a very short list of OTUs, that appear in the jRip rules and are sufficient for predicting the type of sample, feature ranking (ReliefF) in the WEKA software returns a ranking of the complete list of OTUs in terms of their importance. With this third machine learning task, we ranked OTUs according to their relevance for distinguishing between control and *C. difficile* conditioned medium samples. Most of the top 20 relevant OTUs identified by WEKA ReliefF were also identified by the mothur analysis. These included *Escherichia_Shigella*, *Bacteroides*, *Clostridium_XIVa*, *Enterococcus*, *Clostridium_sensu_stricto*, *Streptococcus*, *Coprococcus*, *Lactobacillus* and an unclassified member of *Ruminococcaceae* (Table 1).

Several OTUs also significantly differed between the two ribotypes. LEfSe and PCT have agreed that the following OTUs are associated with ribotype 027: *Escherichia_Shigella*, *Phascolarctobacterium*, *Clostridium_XIVa*, *Dorea* and *Clostridium*_*XVIII* (Fig. 4). On the other hand, ribotype 014/020 was associated with an increased proportion of *Bacteroides*, *Clostridium_XIVa*, *Flavonifractor*, *Coprococcus* and an unclassified taxon from *Ruminococcaceae* (Fig. 4). Each approach also detected some additional OTUs that differed between the two ribotypes.

### Changes in fecal microbiota after growth in *C. difficile* conditioned medium are associated with nutrient availability

To partially address the possible mechanisms underlying the observed changes in fecal microbiota cultivated in *C. difficile* conditioned media the growth of selected representatives in pure culture in conditioned medium was tested (Fig. S3). Of all tested strains the growth in conditioned medium was mostly affected for *Bifidobacterium longum*, *E. coli* and *Ruminococcus gnavus* and less so for *Bacteroides vulgatus* and *Enterococcus faecium*. To rule out direct inhibitory effect of *C. difficile*, the overlapping drop assay was performed on solid media. No direct inhibition, except partially for *R. gnavus*, was observed (Fig. S4).

### Changes in fecal microbiota after growth in conditioned medium are *C. difficile* specific

As the original pooled fecal material used in the experiments described in the previous sections was no longer available, new fecal starting material was prepared for subsequent experiments. The first fecal sample was prepared by pooling specimens from healthy donors before antibiotic therapy and the second fecal sample by pooling specimens from the same individuals after antibiotic therapy.

Changes in both types of fecal microbiota (healthy and post-antibiotic) after cultivation in *C. difficile* conditioned medium and conditioned media of other bacteria (*E. coli, S*. Enteritidis*, Staphylococcus epidermidis*) were compared to cultivation in un-conditioned medium.

**Figure 5:**
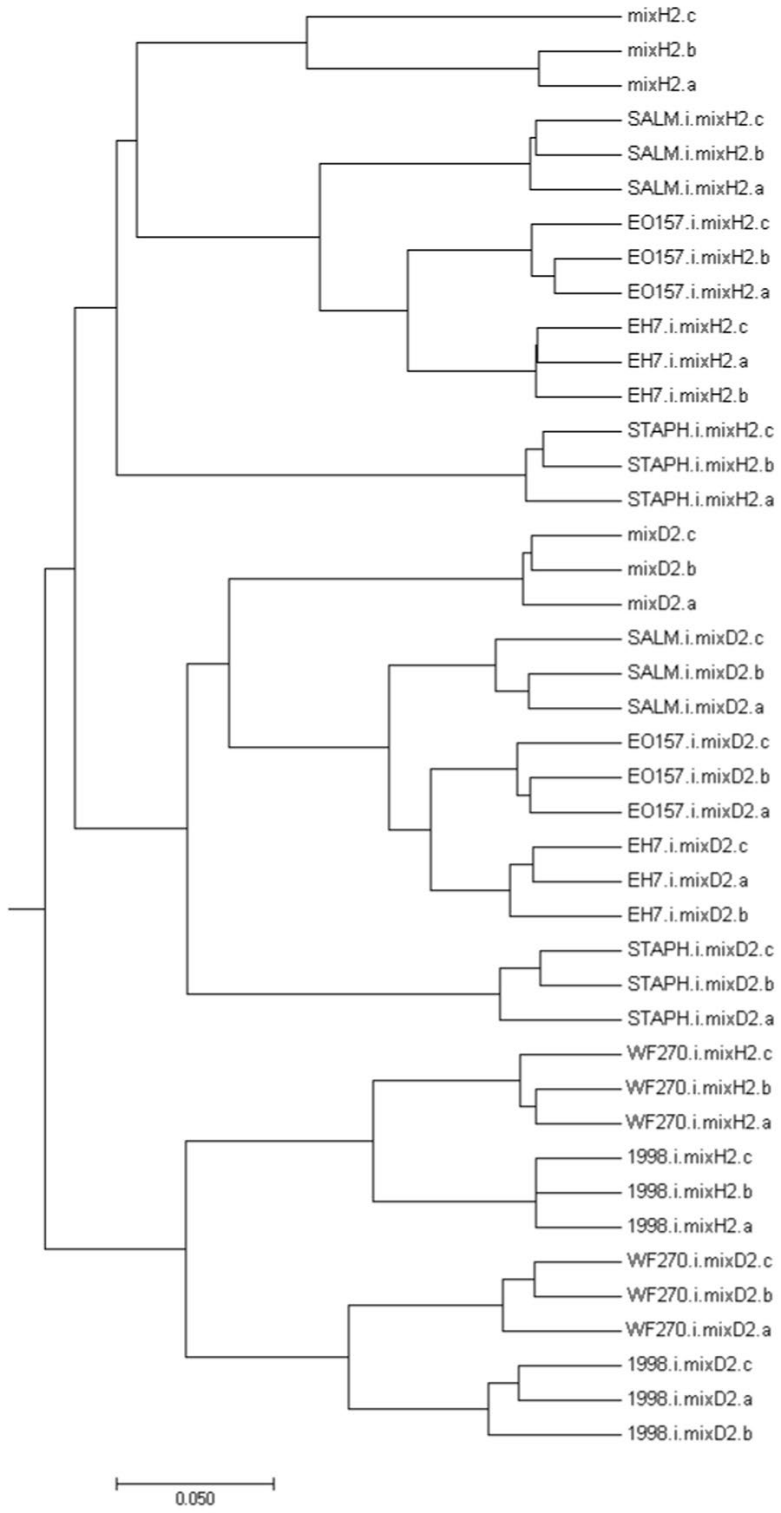
Hierarchical clustering of samples before antibiotic therapy (mixH2) and after antibiotic therapy (mixD2) cultured in WCAB (Wilkins Chalgren Anaerobe Broth) conditioned media of four different bacteria (*Clostridium difficile*, 1998 and WF270; *E. coli*, EH7 and EO157; *Salmonella* Enteritidis, SALM; *Staphylococcus epidermidis*, STAPH), obtained by MEGA software (v.7.0.26).

Changes detected in fecal microbiota composition showed significant differences after growth in all conditioned media (Fig. 5; Fig. S5). The deepest branching divided samples of *C. difficile* conditioned media from all other samples (Fig. 5). Controls from unconditioned medium were more similar to conditioned medium of all other bacteria than to conditioned medium of *C. difficile*. Within *C. difficile* samples, further clustering was associated with fecal microbiota type (healthy or post-antibiotic) and further with *C. difficile* ribotype. Also, in the group of samples from all other bacteria, the first branching was microbiota type associated (healthy or post-antibiotic). Subsequently, all three typical enteric strains clustered together, while fecal microbiota from *S. epidermidis* conditioned medium clustered separately.

## Discussion

*C. difficile* colonizes the gut only in the presence of non-mature or non-functional microbiota. The expanded *C. difficile* population could subsequently shape the gut microenvironment and affect gut microbiota reestablishment, but this possibility is poorly explored. In the present study, we have mimicked the conditions of a *C. difficile* influenced environment by comparing fecal microbiota community structure after cultivation in *C. difficile* conditioned and fresh growth medium.

Possible mechanisms of interactions between pathobionts and gut microbiota include nutrient depletion, direct inhibition, and effect via immune defenses^38^. Effects observed in experiments with conditioned medium could be the result of changes in nutrients or inhibitory substances. In co-culture of *C. difficile* and selected bacterial species, no direct inhibition was detected, except for *R. gnavus*, while all tested strains showed various degrees of growth decrease in *C. difficile* conditioned medium (Fig. S3). Hence, we concluded that poor growth in *C. difficile* conditioned medium is more likely due to changed nutrient availability than to inhibitory substances potentially produced by *C. difficile* and the same mechanisms could also be effective *in vivo* during CDI.

Growth medium conditioned by any bacterial species has modified composition and is likely nutrient depleted and will therefore affect the composition of microbiota after cultivation. To test whether changes in microbiota grown in *C. difficile* conditioned medium are specific, we compared two different types of pooled fecal microbiota (pre- and post-antibiotics) after cultivation in *C. difficile* conditioned medium with conditioned media of other bacteria. *E.coli* and *Salmonella* were selected as representatives of gut pathogens/commensals, whereas *S. epidermidis* was selected as an example of a bacterium not associated with the gut environment. Interestingly, microbiota in *E. coli* and *Salmonella* conditioned medium were more similar to microbiota in *Staphylococcus* conditioned medium than to *C. difficile*. The specific nature of changes was to some extent confirmed also in gnotobiotic mouse model with simplified 12 species murine microbiota (Oligo-MM^12^), where colonization with *C. difficile* (but not with *C. scindens*) resulted in significant compositional perturbation as compared to uninfected control animals^28^. The exact changes were not described in detail, as the main focus of the study was bile acid metabolism in association with colonization resistance.

Cultivation of pooled fecal sample in *C. difficile* conditioned medium decreased microbial diversity and changed OTU distribution. The decreased diversity is in accordance with the majority of studies on human fecal samples, where lower microbial diversity in *C. difficile* positive samples was detected by culture or molecular approaches^7,30,39–48^. Although the association of *C. difficile* colonization with a decrease in gut microbiota diversity is well known, our results additionally show, that it could be explained as a cause, as well as a consequence, of CDI. CDI is initially caused by antibiotics or other factors and allows *C. difficile* overgrowth. Our results suggest that subsequently decreased microbiota diversity is to some extent maintained by *C. difficile* during CDI.

In some studies, ribotype 027 has been reported to be associated with less diverse microbiota^7,30,31^. However, in our *in vitro* model, only fecal microbiota composition, but not microbial diversity, differed between ribotypes 027 and 014/020.

Overall, both analysis approaches, mothur and machine learning, showed that similar genera were affected in conditioned media of both *C. difficile* ribotypes. The most prominently influenced OTUs have well defined roles within gut microbiota functions and/or have been previously associated with presence or absence of *C. difficile (Dorea*, *Enterococcus*, *Clostridium_XIVa*, *Veillonella*, *Escherichia_Shigella* and *Lactobacillus*).

The microbiota in *C. difficile* conditioned medium generally contained significantly over-represented opportunistic pathogens, particularly *Enterococcus* members. This is in agreement with several other studies that found association between *C. difficile* colonization/infection and enterococci^44,49^ or vancomycin-resistant enterococci (VRE)^50–53^. VRE colonization was also described as a risk factor for CDI recurrence^51,52^. Another opportunistic pathogen, *Escherichia_Shigella*, was found in reduced proportion in *C. difficile* conditioned medium. Reduced growth was also observed for pure cultures of *E. coli* in *C. difficile* conditioned medium, while *E. faecium* was affected to a much lesser degree (Fig. S3).

On the other hand, in *C. difficile* conditioned medium we found a significant under-representation of gut commensals with putative protective functions, particularly lactate (*Lactobacillus*) and butyrate (*Clostridium_XIVa*) producers. Some previous studies already reported on lower lactobacilli counts in *C. difficile* positive samples^39,42^. Our findings suggest that lactobacilli, an important bacterial group for balanced microbiota, regenerate poorly in *C. difficile* presence. Poor growth of lactobacilli in the presence of *C. difficile* could also explain why some probiotics have low efficiency in the prevention of CDI in several clinical trials^54,55^. Nevertheless, the probiotic strain *Lactobacillus plantarum 299v* ^56,57^, a mixture of *Lactobacillus* probiotic strains^58^ or lactobacilli mixed with other probiotic strains^59^ could have strong potential as adjunctive therapies to antibiotics for the prevention of CDI. Due to a decreased abundance of several butyrate-producing bacteria, including *Clostridium_XIVa* members, in *C. difficile* positive samples^43^ and their re-establishment after fecal microbiota transplantation^60,61^, there is growing evidence about the important role of those bacteria in colonization resistance to CDI.

Furthermore, in *C. difficile* conditioned media, we found a decreased proportion of some other putative butyrate producing bacteria, belonging to the family *Lachnospiraceae*, i.e. *Blautia* and *Dorea*. This correlates with studies on humans and animal models showing association of both genera with colonization resistance to CDI^12,62^. In addition to their different relative abundance between *C. difficile* conditioned medium (any ribotype) and control medium, both genera (together with *Bacteroides*), were among those that differentiated between ribotype 027 and ribotype 014/020 exposed fecal microbiota in our *in vitro* model. Some *Dorea* OTUs showed relative increase and other decrease between the *C. difficile* conditioned and control media. This could correspond to different functions associated with the genus. Although *Dorea* is a genus with recognised positive effects on gut and human health, increased proportions of *Dorea* have previously also been associated with colorectal adenomas^63^ and the irritable bowel syndrome^64^.

Another affected genus was *Veillonella*, which showed decrease in relative abundance in *C. difficile* conditioned medium. To date, the reports on *Veillonella* and CDI are somewhat conflicting. A recent study by Antharam and co-workers (2016) linked two *Veillonella* species with specific changes in the gut metabolome that correlated strongly with CDI^9^. Patients with recurrent CDI possessed an increased proportion of *Veillonella* ^62^, while oncological patients colonized with *C. difficile* had decreased levels of *Veillonella* ^65^.

In summary, we could show that *C. difficile* causes specific changes in fecal microbiota and that nutrient availability is a more likely mechanism than direct inhibitory effects. Changes impact common gut microbiota members associated with colonization resistance or are associated with an increased proportion of opportunistic microorganisms. The two tested *C. difficile* ribotypes differ in their impact on fecal microbiota composition. Our results suggest that decreased microbiota diversity initially caused by antibiotics and predisposing to CDI, is to some extent maintained by *C. difficile* during and after the infection.

## Methods

### Conditioned medium preparation

Six different *C. difficile* strains, belonging to two PCR ribotypes (014/020, 027), were selected from our *C. difficile* strain collection (strain designations ZZV10-2514, ZZV11-3188, ZZV11-3298, ZZV11-3304, ZZV12-4777, ZZV14-5907). Selected strains originated from humans and were isolated between the years 2010 and 2014. All strains were incubated in anaerobic workstation at 37 °C (Don Whitley Scientific) for 48 hours in Wilkins Chalgren Anaerobe Broth (WCAB) (Oxoid) and Anaerobe Basal Broth (ABB) (Oxoid) to obtain *C. difficile* suspensions with up to 10^10^ CFU/ml (WCAB) and up to 10^9^ CFU/ml (ABB), respectively. After centrifugation at 10,000 rpm for 5 min, supernatants were filtered (0.2 μm, cellulose acetate, Sarstedt) to prepare conditioned medium, which was subsequently used for fecal microbiota culturing.

### *In vitro* model of *C. difficile* conditioned medium and fecal microbiota

*C. difficile* negative fecal samples were randomly selected from samples sent for *C. difficile* testing (male: n = 5; female: n = 5, all aged under 65 years). Specimens were pooled and diluted in glycerol to form 10% slurry and immediately frozen at −80 °C until further processing. Fecal slurry was added to growth media (ABB, WCAB) (1:100, v:v) and incubated overnight to prepare fecal inoculum for *in vitro* cultivation. This overnight fecal inoculum (50 μl) was added into ABB or WCAB conditioned medium (4,950 μl) in a 6-well plate. Cultures were gently mixed and then incubated anaerobically at 37 °C for five days. Samples (5 ml) for total bacterial DNA extraction were taken after 3 and 5 days of incubation. For each sample with a specific combination of growth medium, *C. difficile* strain and incubation time, three parallels were tested. Fecal inoculum grown in freshly prepared and prereduced ABB and WCAB growth media was used as a control. All incubations and sample handling were performed anaerobically at 37 °C in an anaerobic workstation (10% CO2, 10% H2, 80% N2) (Don Whitley Scientific).

### Cultivation of fecal microbiota *in C. difficile* conditioned medium in comparison to media conditioned by other bacteria

This set of experiments was performed as described above but using only WCAB medium, different inoculum of pooled fecal microbiota, only two *C. difficile* strains and additional other bacteria.

Besides two *C. difficile* strains (ZZV11-3298, ZZV14-5907) we tested *Salmonella* Enteritidis strain (ATCC 13076), *Staphylococcus epidermidis* strain (ATCC 12228) and two *E. coli* strains. One of them was O157 strain isolated in diagnostic laboratory (ZZV17-8567) and an isolate obtained from fecal sample used also for inoculum preparation (ZZV17-8330). Strains were incubated anaerobically or aerobically and conditioned medium was prepared as described above.

Two different fecal inoculums were prepared by pooling fecal samples of three donors (male: n = 1, female: n = 2, all aged under 65 years). Inoculum designated ‘mixH2’ was prepared from samples obtained before antibiotic therapy (clarithromycin, penicillin V or amoxicillin). Inoculum designated ‘mixD2’ was prepared from samples obtained from same individuals three days after completed antibiotic therapy.

Cultures were gently mixed and incubated anaerobically at 37 °C for three days. For each sample with a specific combination of growth medium, bacterial strain and type of microbiota, three parallels were tested.

### Cultivation of pure cultures of selected bacteria in *C. difficile* conditioned media or in co-culture with *C. difficile*

*Bifidobacterium longum* (ZZV17-8523), *Bacteroides vulgatus* (ZZV17-8401), *Enterococcus faecium* (ZZV17-8382), *E. coli* (ZZV17-8330) and *Ruminococcus gnavus* (ZZV11-4196) were grown anaerobically at 37 °C in fresh WCAB medium and in conditioned WCAB medium of two *C. difficile* strains (ZZV11-3298, ZZV14-5907). For each condition, the experiment was done in triplicates. Growth was monitored by OD_620_ measurement (Synergy2, BioTek Instruments) at different time points (0 hr, 4 hr, 8 hr, 12 hr, 24 hr, 48 hr and 72 hr).

The same isolates were co-cultured with the same two *C. difficile* strains on Wilkins Chalgren Anaerobe Agar (WCAA). We used the overlapping drop technique as described by Parker and Simmons (1959)^66^ with minor modifications. Pairs of overlapping drops of overnight WCAB broth cultures were placed on WCAA solid medium. Drops of 7 µl were deposited on WCAA plate: the second drop of each pair was added as soon as the first had dried and overlapped the first by approximately 1/3 of its area. Plates were examined for inhibition extending beyond the area of overlap after 24 hours of anaerobic incubation at 37 °C.

### Isolation of the total bacterial DNA and high-throughput 16S rDNA amplicon sequencing

Samples were centrifuged at 10,000 rpm for 10 min and the pellet was used for total bacterial DNA isolation (QIAamp Fast Stool DNA Mini Kit, Qiagen) after mechanical disruption (speed 7,000 for 70 s) with the SeptiFast Lyse Kit (Roche) on MagNA Lyser (Roche). Bacterial community composition was determined by paired-end sequencing on an Illumina MiSeq platform, targeting the V3-V4 hypervariable region of the 16 S rRNA gene. Library preparation was carried out using the 341 F (5′-CCTACGGGNGGCWGCAG-3′) – 805 R (5′-GACTACHVGGGTATCTAATCC-3′) set of primers. Sequencing yielded an average depth of 140,000 sequences per sample. A template-free sample processed in the same way as the real samples and sequenced on the same run was included as negative sequencing control.

### Data analysis

MiSeq output data was analysed with statistical tools included in the mothur software and different machine learning approaches.

Analysis in mothur (v.1.36.1)^67^ was done according to the MiSeq standard operating procedure (SOP) for Illumina paired end reads^68^. The following criteria were included in the analysis: i) sequences were not allowed any ambiguous bases and the maximum homopolymer length was set to 8 base pairs; ii) sequences were aligned against the Silva reference alignment; iii) chimeras were searched with the UCHIME algorithm; iv) classification of sequences was performed using the RDP training set (v.9) with 0.80 threshold value; v) OTUs (operational taxonomic units) were constructed with 0.03 cutoff value; vi) OTUs with relative abundance lower than 0.001% were removed from contingency table. Alpha sample diversity was calculated with the Shannon diversity index. Beta diversity was estimated with the AMOVA test, which was performed using Bray Curtis distances^69^. OTUs that differed between treatments were selected with respect to linear discriminant analysis (LDA) effect size (LEfSe) method^33^. Non-Metric Multidimensional Scaling (NMDS) analysis was done in R with the vegan package using the Bray Curtis distance matrix as input. Hierarchical clustering was obtained with MEGA software (v.7.0.26)^70^ using the Bray Curtis distance matrix as input.

We applied three types of machine learning methods to analyze the collected data. In machine learning terminology, each data sample is called an example and corresponds to a row in the data table. The variables, also called attributes, correspond to columns in the data table. We distinguish between descriptive variables (inputs) and target variables (outputs). In the first machine learning task in this study, we used incubation period and type of sample (control, ribotype 014/020 and ribotype 027) as descriptive variables and the bacterial community composition in terms of the relative abundance of OTUs as target variables. In the second and third tasks, we used the relative abundances of OTUs as description of the type of sample as the target variable.

The different types of machine learning methods we applied were: learning predictive clustering trees (PCTs)^35^, learning classification rules (RIPPER)^36^ and feature ranking (ReliefF)^34^. In particular, we used the software packages CLUS (available at: http://sourceforge.net/projects/clus/)^35^ for learning PCTs and WEKA v3.8 (Waikato Environment for Knowledge Analysis) for learning rules (jRip implementation of RIPPER) and feature ranking^37^.

## Electronic supplementary material

Supplementary Information
