# Supplementary material for: Evaluating the effect of Clostridium difficile conditioned medium on fecal microbiota community structure
Source: Sci Rep. 2017 Nov 27;7:16448. doi: 10.1038/s41598-017-15434-1 (PMC5703886; doi:10.1038/s41598-017-15434-1)
Supplement: Supplementary file 1 — Supplementary Information [file 41598_2017_15434_MOESM1_ESM.doc]

**Supplementary Information**

**Evaluating the effect of *Clostridium difficile* conditioned medium on fecal microbiota community structure**

Sabina Horvat^1^, Aleksander Mahnic^2^, Martin Breskvar^3,4^, Saso Dzeroski^3,4,5^, and Maja Rupnik^1,2*^

^1^University of Maribor, Faculty of Medicine, Maribor, Slovenia

^2^National Laboratory for Health, Environment and Food, Maribor, Slovenia

^3^Jozef Stefan Institute, Ljubljana, Slovenia

^4^Jozef Stefan International Postgraduate School, Ljubljana, Slovenia

^5^Centre of Excellence for Integrated Approaches in Chemistry and Biology of Proteins, Ljubljana, Slovenia

^*^maja.rupnik@nlzoh.si

**Table S1**: The composition of fecal microbiota on genus level in pooled faecal sample before any culturing (point 0) and comparison with microbiota on day 3 and day 5 in Wilkins Chalgren Anaerobe Broth (WCAB) and Anaerobe Basal Broth (ABB) control medium. Only genera with relative abundance larger than 1% are presented.

|  |  | WCAB | | ABB | |
| --- | --- | --- | --- | --- | --- |
|  | 0 | 3 | 5 | 3 | 5 |
| *Bifidobacterium* | 0.0264 | 0.0014 | 0.0010 | 0.0013 | 0.0019 |
| *Bacteroides* | 0.2956 | 0.1959 | 0.1974 | 0.1890 | 0.1551 |
| *Prevotella* | 0.0591 | 0.0000 | 0.0000 | 0.0000 | 0.0000 |
| *Alistipes* | 0.0246 | 0.0045 | 0.0074 | 0.0068 | 0.0119 |
| *Parabacteroides* | 0.0155 | 0.0136 | 0.0088 | 0.0197 | 0.0125 |
| *Barnesiella* | 0.0102 | 0.0036 | 0.0057 | 0.0071 | 0.0066 |
| *Escherichia_Shigella* | 0.1593 | 0.2076 | 0.1710 | 0.1289 | 0.1049 |
| *Gemmiger* | 0.0119 | 0.0000 | 0.0000 | 0.0000 | 0.0000 |
| *Sutterella* | 0.0051 | 0.0281 | 0.0338 | 0.0351 | 0.0414 |
| *Blautia* | 0.0364 | 0.0104 | 0.0131 | 0.0049 | 0.0044 |
| *Clostridium_sensu_stricto* | 0.0015 | 0.0669 | 0.0464 | 0.0013 | 0.0003 |
| *Streptococcus* | 0.0083 | 0.0428 | 0.0351 | 0.0351 | 0.0045 |
| *Dorea* | 0.0048 | 0.0352 | 0.0439 | 0.0194 | 0.0275 |
| *Peptostreptococcus* | 0.0001 | 0.0037 | 0.0183 | 0.0254 | 0.0906 |
| *Faecalibacterium* | 0.0774 | 0.0010 | 0.0007 | 0.0220 | 0.0054 |
| *Dialister* | 0.0167 | 0.0006 | 0.0003 | 0.0002 | 0.0001 |
| *Veillonella* | 0.0157 | 0.1450 | 0.1974 | 0.1564 | 0.1450 |
| *Ruminococcus* | 0.0127 | 0.0000 | 0.0000 | 0.0000 | 0.0000 |
| *Acidaminococcus* | 0.0000 | 0.0606 | 0.0519 | 0.0820 | 0.0632 |
| *Lachnospiracea_incertae_sedis* | 0.0241 | 0.0001 | 0.0001 | 0.0000 | 0.0000 |
| *Roseburia* | 0.0205 | 0.0016 | 0.0011 | 0.0000 | 0.0000 |
| *Clostridium_XlVa* | 0.0038 | 0.0401 | 0.0392 | 0.1324 | 0.1613 |
| *Phascolarctobacterium* | 0.0007 | 0.0383 | 0.0408 | 0.0440 | 0.0560 |
| *Enterococcus* | 0.0001 | 0.0182 | 0.0154 | 0.0145 | 0.0178 |
| *Oscillibacter* | 0.0020 | 0.0123 | 0.0068 | 0.0003 | 0.0010 |
| *Flavonifractor* | 0.0012 | 0.0089 | 0.0108 | 0.0065 | 0.0096 |
| unclassified | 0.0792 | 0.0250 | 0.0200 | 0.0444 | 0.0533 |

**Table S2:** Specific bacterial populations clearly differentiate freshly prepared control growth media from conditioned media of ribotypes 014/020 and 027. The LEfSe test (mothur software) was used to compare control (C) and conditioned medium of two ribotypes (014/020, 027), on day 3 and 5. OTUs with negative LDA scores (red) are enriched in control samples, while OTUs with positive LDA scores (blue) are enriched in samples of given ribotype. To compare conditioned medium of ribotype 014/020 and conditioned medium of ribotype 027 with each other, negative LDA scores (red) are used to represent OTUs that are enriched in conditioned medium of ribotype 014/020 and positive LDA scores (blue) represent OTUs that are enriched in conditioned medium of ribotype 027.

| medium | WCAB (Wilkins Chalgren Anaerobe Broth) | | | | | |  |  |  |
| --- | --- | --- | --- | --- | --- | --- | --- | --- | --- |
| day | 3 | 5 | 3 | 5 | 3 | 5 |  |  |  |
| comparison | **C-014** | **C-014** | **C-027** | **C-027** | **014-027** | **014-027** |  |  |  |
| AMOVA | 0.002 | <0.001 | 0.003 | 0.004 | 0.043 | 0.001 | Phylum | Genus | Relative abundance |
| Otu00001 | -4.76 | -4.63 | -4.75 | -4.60 | 0.00 | 3.50 | Proteobacteria | Escherichia_Shigella | 0.044651 |
| Otu00002 | 4.39 | 4.36 | 4.35 | 4.34 | 0.00 | 0.00 | Bacteroidetes | Bacteroides | 0.063761 |
| Otu00003 | -4.21 | -4.58 | -4.23 | -4.59 | 0.00 | 0.00 | Firmicutes | Veillonella | 0.052010 |
| Otu00004 | 0.00 | 0.00 | 0.00 | 0.00 | 0.00 | 0.00 | Firmicutes | Acidaminococcus | 0.024833 |
| Otu00005 | 4.18 | 4.14 | 4.13 | 4.04 | 0.00 | -3.53 | Bacteroidetes | Bacteroides | 0.026268 |
| Otu00006 | 3.61 | 0.00 | 3.84 | 0.00 | 3.48 | 3.69 | Firmicutes | Phascolarctobacterium | 0.018883 |
| Otu00007 | 4.10 | 4.13 | 4.06 | 4.04 | 0.00 | -3.57 | Firmicutes | Clostridium_XlVa | 0.015607 |
| Otu00008 | 4.06 | 4.19 | 4.09 | 4.29 | 0.00 | 0.00 | Firmicutes | Dorea | 0.019565 |
| Otu00009 | -3.49 | -3.51 | -3.49 | -3.53 | 0.00 | 0.00 | Bacteroidetes | Bacteroides | 0.009910 |
| Otu00010 | 0.00 | 0.00 | 3.86 | 0.00 | 3.52 | 0.00 | Firmicutes | Peptostreptococcus | 0.007472 |
| Otu00011 | 3.82 | 3.80 | 3.80 | 0.00 | 0.00 | 0.00 | Proteobacteria | Sutterella | 0.016825 |
| Otu00012 | 4.04 | 3.96 | 4.01 | 3.95 | 0.00 | 0.00 | Firmicutes | Enterococcus | 0.014120 |
| Otu00013 | -3.28 | -3.12 | -3.16 | 0.00 | 2.58 | 2.91 | Firmicutes | Clostridium_XlVa | 0.003472 |
| Otu00014 | 3.58 | 4.03 | 3.52 | 4.05 | 0.00 | 0.00 | Firmicutes | unclassified from Lachnospiraceae | 0.006143 |
| Otu00015 | 3.04 | 3.23 | 3.02 | 3.04 | 0.00 | -2.78 | Firmicutes | unclassified from Ruminococcaceae | 0.001368 |
| Otu00016 | 3.61 | 3.68 | 3.62 | 3.67 | 0.00 | 0.00 | Bacteroidetes | Parabacteroides | 0.006223 |
| Otu00017 | 4.03 | 3.48 | 4.03 | 0.00 | 0.00 | -3.19 | Bacteroidetes | Bacteroides | 0.007750 |
| Otu00018 | -4.50 | -4.34 | -4.50 | -4.35 | -2.58 | -2.32 | Firmicutes | Clostridium_sensu_stricto | 0.004062 |
| Otu00019 | -4.11 | -4.07 | -4.10 | -4.07 | 0.00 | 0.00 | Firmicutes | Streptococcus | 0.006720 |
| Otu00020 | 3.62 | 3.75 | 3.49 | 3.54 | -3.19 | -3.29 | Firmicutes | Flavonifractor | 0.007102 |
| Otu00021 | 3.35 | -3.35 | 3.52 | -3.16 | 3.07 | 0.00 | Firmicutes | Blautia | 0.005151 |
| Otu00022 | -3.99 | -3.70 | -3.94 | -3.63 | 3.16 | 2.78 | Firmicutes | Dorea | 0.002549 |
| Otu00023 | 2.73 | 0.00 | 3.01 | 3.09 | 2.62 | 2.78 | Firmicutes | Clostridium_XVIII | 0.002431 |
| Otu00024 | 0.00 | 3.10 | 0.00 | 0.00 | 0.00 | 0.00 | Actinobacteria | unclassified from Coriobacteriaceae | 0.003454 |
| Otu00025 | 3.60 | 3.62 | 3.56 | 3.58 | 0.00 | 0.00 | Bacteroidetes | Bacteroides | 0.004139 |
| Otu00026 | 0.00 | 0.00 | 0.00 | 0.00 | 0.00 | 0.00 | Firmicutes | Clostridium_XlVa | 0.004277 |
| Otu00027 | 3.99 | 3.88 | 3.86 | 3.73 | -3.44 | -3.33 | Firmicutes | Coprococcus | 0.005436 |
| Otu00028 | 2.97 | 2.65 | 0.00 | 2.41 | -2.83 | 0.00 | Firmicutes | unclassified from Lachnospiraceae | 0.000636 |
| Otu00029 | 0.00 | 0.00 | 0.00 | 0.00 | 0.00 | 0.00 | Firmicutes | unclassified from Ruminococcaceae | 0.004383 |
| Otu00030 | -2.86 | -2.63 | -2.89 | -2.58 | 0.00 | 0.00 | Bacteroidetes | Parabacteroides | 0.001307 |
| Otu00031 | 0.00 | 0.00 | 0.00 | 0.00 | 2.99 | 0.00 | Bacteroidetes | Barnesiella | 0.001856 |
| Otu00032 | -2.61 | -2.50 | -2.63 | -2.38 | 0.00 | 0.00 | Firmicutes | Faecalibacterium | 0.000117 |
| Otu00033 | 0.00 | 0.00 | 0.00 | 0.00 | 0.00 | 0.00 | Firmicutes | Eubacterium | 0.003375 |
| Otu00034 | 3.25 | 3.48 | 3.29 | 3.54 | 0.00 | 0.00 | Firmicutes | unclassified from Ruminococcaceae | 0.001930 |
| Otu00035 | -3.01 | 0.00 | -2.96 | -2.87 | 0.00 | 0.00 | Bacteroidetes | Alistipes | 0.001558 |
| Otu00036 | 0.00 | 0.00 | 0.00 | 0.00 | 0.00 | 0.00 | Bacteroidetes | Bacteroides | 0.001088 |
| Otu00037 | 2.96 | 0.00 | 0.00 | 0.00 | -2.81 | 0.00 | Firmicutes | unclassified from Lachnospiraceae | 0.001799 |
| Otu00038 | 0.00 | -3.33 | 0.00 | -3.17 | 0.00 | 0.00 | Firmicutes | Oscillibacter | 0.002318 |
| Otu00039 | 2.92 | 2.68 | 2.88 | 2.55 | -2.48 | -2.42 | Bacteroidetes | Bacteroides | 0.000917 |
| Otu00040 | 2.80 | 2.80 | 2.86 | 2.77 | 0.00 | 0.00 | Actinobacteria | Collinsella | 0.000663 |
| Otu00041 | 3.08 | 3.29 | 2.98 | 3.25 | -2.49 | 0.00 | Bacteroidetes | Bacteroides | 0.001544 |
| Otu00042 | -2.25 | 0.00 | 0.00 | -2.06 | 0.00 | 0.00 | Firmicutes | Butyricicoccus | 0.000382 |
| Otu00043 | 2.30 | 2.94 | 2.56 | 2.91 | 0.00 | 0.00 | Actinobacteria | Eggerthella | 0.000679 |
| Otu00044 | 0.00 | 2.39 | 0.00 | 0.00 | 0.00 | 0.00 | Firmicutes | Clostridium_XlVa | 0.000629 |
| Otu00045 | 2.89 | 2.94 | 3.01 | 2.92 | 0.00 | 0.00 | Proteobacteria | unclassified from Burkholderiales | 0.001010 |
| Otu00046 | 2.74 | 2.67 | 2.69 | 2.66 | 0.00 | 0.00 | Bacteroidetes | Bacteroides | 0.000604 |
| Otu00047 | 0.00 | 2.04 | 0.00 | 0.00 | 0.00 | 0.00 | Actinobacteria | Bifidobacterium | 0.000423 |
| Otu00048 | 0.00 | 0.00 | 0.00 | -2.33 | 0.00 | 0.00 | Firmicutes | Streptococcus | 0.000651 |
| Otu00049 | 0.00 | 0.00 | 0.00 | 0.00 | 0.00 | 0.00 | Proteobacteria | Parasutterella | 0.000623 |
| Otu00050 | 2.91 | 2.82 | 2.71 | 2.74 | -2.51 | 0.00 | Firmicutes | Erysipelotrichaceae_incertae_sedis | 0.000551 |
| Otu00051 | 2.12 | 2.16 | 0.00 | 0.00 | 0.00 | 0.00 | unclassified | unclassified | 0.000301 |
| Otu00052 | 0.00 | 2.26 | 0.00 | 0.00 | 0.00 | 0.00 | Firmicutes | Oscillibacter | 0.000217 |
| Otu00053 | 2.51 | 0.00 | 2.26 | 2.56 | 0.00 | 0.00 | Firmicutes | unclassified from Ruminococcaceae | 0.000496 |
| Otu00054 | 2.19 | 2.48 | 0.00 | 2.34 | 0.00 | 0.00 | Firmicutes | Holdemania | 0.000221 |
| Otu00055 | 2.73 | 0.00 | 0.00 | -2.45 | 0.00 | 0.00 | Proteobacteria | Sutterella | 0.000370 |
| Otu00056 | 0.00 | 3.09 | 0.00 | 3.00 | 0.00 | 0.00 | Firmicutes | unclassified from Clostridiales | 0.000464 |
| Otu00057 | 0.00 | 0.00 | 0.00 | 2.02 | -2.02 | 0.00 | unclassified | unclassified | 0.000084 |
| Otu00058 | 0.00 | 0.00 | 0.00 | 0.00 | 0.00 | 0.00 | Firmicutes | Clostridium_XlVa | 0.000184 |
| Otu00059 | -3.27 | -3.04 | -3.30 | -3.12 | 0.00 | 0.00 | Firmicutes | Lactobacillus | 0.000304 |
| Otu00060 | -2.31 | -2.30 | -2.42 | -2.42 | 0.00 | 0.00 | Firmicutes | Roseburia | 0.000379 |
| Otu00061 | 0.00 | 2.34 | 2.35 | 2.48 | 2.17 | 0.00 | Firmicutes | Dialister | 0.000314 |
| Otu00062 | 2.31 | 2.32 | 0.00 | 0.00 | 0.00 | 0.00 | Firmicutes | Clostridium_sensu_stricto | 0.000319 |
| Otu00063 | 0.00 | 0.00 | -2.18 | 0.00 | 0.00 | 0.00 | Proteobacteria | Raoultella | 0.000122 |
| Otu00064 | 0.00 | 0.00 | -2.35 | 0.00 | 0.00 | 0.00 | Firmicutes | Lactobacillus | 0.000210 |
| Otu00065 | -2.94 | -2.81 | -2.95 | -2.80 | 0.00 | 0.00 | Firmicutes | unclassified from Ruminococcaceae | 0.000229 |
| Otu00066 | -2.21 | -2.57 | 0.00 | 0.00 | 0.00 | 2.20 | Firmicutes | Coprococcus | 0.000247 |
| Otu00067 | -2.43 | -2.69 | -2.50 | -2.64 | 0.00 | 0.00 | Proteobacteria | Enterobacter | 0.000200 |
| Otu00068 | 0.00 | 0.00 | 0.00 | 0.00 | 0.00 | 0.00 | Firmicutes | unclassified from Ruminococcaceae | 0.000093 |
| Otu00070 | 0.00 | -2.72 | 0.00 | -2.59 | 0.00 | 0.00 | Bacteroidetes | Alistipes | 0.000116 |
| Otu00071 | 0.00 | 0.00 | -2.19 | -2.22 | 0.00 | 0.00 | Proteobacteria | unclassified from Enterobacteriaceae | 0.000096 |
| Otu00072 | 0.00 | -2.20 | -2.39 | -2.58 | -2.05 | -2.03 | Proteobacteria | Citrobacter | 0.000119 |
| Otu00073 | 0.00 | 0.00 | 0.00 | 0.00 | 0.00 | 0.00 | Firmicutes | Erysipelotrichaceae_incertae_sedis | 0.000110 |
| Otu00074 | 2.09 | 0.00 | 2.23 | 0.00 | 0.00 | 0.00 | Firmicutes | Lachnospiracea_incertae_sedis | 0.000067 |
| Otu00075 | 0.00 | 0.00 | 0.00 | 0.00 | 0.00 | 0.00 | Firmicutes | Lactococcus | 0.000100 |
| Otu00076 | -2.32 | -2.29 | -2.33 | -2.36 | 0.00 | 0.00 | Bacteroidetes | Bacteroides | 0.000044 |
| Otu00077 | 0.00 | 0.00 | 0.00 | 0.00 | 0.00 | 0.00 | Actinobacteria | Bifidobacterium | 0.000119 |
| Otu00078 | 0.00 | 0.00 | 0.00 | 0.00 | 0.00 | 0.00 | Firmicutes | unclassified from Ruminococcaceae | 0.000038 |
| Otu00079 | 0.00 | 0.00 | 0.00 | 2.03 | 0.00 | 0.00 | Firmicutes | unclassified from Ruminococcaceae | 0.000020 |
| Otu00080 | 0.00 | 0.00 | 0.00 | 2.43 | 0.00 | 0.00 | Firmicutes | unclassified from Lachnospiraceae | 0.000064 |
| Otu00081 | 0.00 | 0.00 | 0.00 | 0.00 | 0.00 | 0.00 | Firmicutes | unclassified from Lachnospiraceae | 0.000022 |
| Otu00083 | -2.21 | -2.05 | -2.25 | -2.27 | 0.00 | 0.00 | Firmicutes | Erysipelotrichaceae_incertae_sedis | 0.000020 |
| Otu00085 | 0.00 | 0.00 | 0.00 | 0.00 | 0.00 | 0.00 | Firmicutes | unclassified from Lachnospiraceae | 0.000032 |
| Otu00086 | 0.00 | 0.00 | 0.00 | 0.00 | 0.00 | 0.00 | Firmicutes | Flavonifractor | 0.000012 |
| Otu00087 | 0.00 | 0.00 | 0.00 | 0.00 | 0.00 | 0.00 | Bacteroidetes | unclassified | 0.000069 |
| Otu00089 | 0.00 | 0.00 | 0.00 | -2.09 | 0.00 | 0.00 | Bacteroidetes | Odoribacter | 0.000011 |
| Otu00090 | -2.42 | -2.35 | -2.36 | -2.30 | 0.00 | 0.00 | Proteobacteria | Parasutterella | 0.000044 |
| Otu00091 | 0.00 | 0.00 | 0.00 | 0.00 | 0.00 | 0.00 | Proteobacteria | Bilophila | 0.000012 |
| Otu00092 | 0.00 | 0.00 | 0.00 | 0.00 | 0.00 | 0.00 | Firmicutes | Oscillibacter | 0.000049 |
| Otu00093 | 0.00 | 0.00 | 0.00 | 0.00 | 0.00 | 0.00 | Firmicutes | Lachnospiracea_incertae_sedis | 0.000049 |
| Otu00095 | 0.00 | 0.00 | 0.00 | 2.04 | 0.00 | 0.00 | Firmicutes | unclassified from Lachnospiraceae | 0.000044 |
| Otu00096 | -2.33 | -2.47 | -2.34 | -2.52 | 0.00 | 0.00 | Firmicutes | Streptococcus | 0.000029 |
| Otu00097 | 0.00 | 0.00 | 0.00 | 0.00 | 0.00 | 0.00 | Firmicutes | unclassified from Ruminococcaceae | 0.000033 |
| Otu00098 | 0.00 | 0.00 | 0.00 | 0.00 | 0.00 | 0.00 | Actinobacteria | Gordonibacter | 0.000009 |
| Otu00099 | 0.00 | 0.00 | 0.00 | 0.00 | 0.00 | 0.00 | Firmicutes | unclassified from Lachnospiraceae | 0.000036 |
| Otu00100 | 0.00 | 0.00 | 0.00 | 0.00 | 0.00 | 0.00 | Firmicutes | Clostridium_IV | 0.000017 |
| Otu00101 | 0.00 | 0.00 | 0.00 | 0.00 | 0.00 | 0.00 | Bacteroidetes | Butyricimonas | 0.000024 |
| Otu00102 | 0.00 | 0.00 | 0.00 | 0.00 | 0.00 | 0.00 | Bacteroidetes | unclassified from Porphyromonadaceae | 0.000033 |
| Otu00104 | 0.00 | 0.00 | 0.00 | 0.00 | 0.00 | 0.00 | Firmicutes | unclassified from Clostridiales | 0.000022 |
| Otu00105 | 0.00 | 0.00 | 0.00 | 0.00 | 0.00 | 0.00 | Firmicutes | unclassified from Lachnospiraceae | 0.000020 |
| Otu00106 | 0.00 | 0.00 | 0.00 | 2.10 | 0.00 | 0.00 | Firmicutes | Enterococcus | 0.000010 |
| Otu00107 | 0.00 | 0.00 | 0.00 | 0.00 | 0.00 | 0.00 | Firmicutes | Flavonifractor | 0.000021 |
| Otu00109 | 0.00 | 0.00 | 0.00 | 0.00 | 0.00 | 0.00 | Bacteroidetes | Bacteroides | 0.000018 |
| Otu00110 | 0.00 | 0.00 | 0.00 | 0.00 | 0.00 | 0.00 | Firmicutes | unclassified from Ruminococcaceae | 0.000015 |
| Otu00111 | 0.00 | -2.30 | 0.00 | -2.35 | 0.00 | 0.00 | Synergistetes | Pyramidobacter | 0.000010 |
| Otu00112 | 0.00 | 0.00 | 0.00 | 0.00 | 0.00 | 0.00 | Actinobacteria | Bifidobacterium | 0.000014 |
| Otu00113 | 0.00 | 0.00 | 0.00 | 0.00 | 0.00 | 0.00 | Firmicutes | unclassified from Ruminococcaceae | 0.000012 |
| Otu00114 | 0.00 | 0.00 | 0.00 | 0.00 | 0.00 | 0.00 | Bacteroidetes | Butyricimonas | 0.000015 |
| Otu00115 | 0.00 | 0.00 | 0.00 | 0.00 | 0.00 | 0.00 | Bacteroidetes | Butyricimonas | 0.000018 |
| Otu00116 | 0.00 | 0.00 | 0.00 | 0.00 | 0.00 | 0.00 | Firmicutes | unclassified from Lachnospiraceae | 0.000012 |
| Otu00117 | 0.00 | 0.00 | 0.00 | 0.00 | 0.00 | 0.00 | Firmicutes | Enterococcus | 0.000012 |
| Otu00118 | 0.00 | 0.00 | 0.00 | 0.00 | 0.00 | 0.00 | Firmicutes | Phascolarctobacterium | 0.000006 |
| Otu00119 | 0.00 | 0.00 | 0.00 | 0.00 | 0.00 | 0.00 | Firmicutes | Blautia | 0.000011 |
| Otu00122 | 0.00 | 0.00 | 0.00 | 0.00 | 0.00 | 0.00 | Proteobacteria | Escherichia_Shigella | 0.000010 |
| Otu00123 | 0.00 | 0.00 | 0.00 | 0.00 | 0.00 | 0.00 | Firmicutes | unclassified from Lachnospiraceae | 0.000016 |
| Otu00124 | 0.00 | 0.00 | 0.00 | 0.00 | 0.00 | 0.00 | Firmicutes | unclassified from Eubacteriaceae | 0.000017 |
| Otu00125 | 0.00 | 0.00 | 0.00 | 0.00 | 0.00 | 0.00 | Firmicutes | Veillonella | 0.000005 |
| Otu00127 | 0.00 | 0.00 | 0.00 | 0.00 | 0.00 | 0.00 | Bacteroidetes | Butyricimonas | 0.000015 |
| Otu00128 | 0.00 | 0.00 | 0.00 | 0.00 | 0.00 | 0.00 | Bacteroidetes | Bacteroides | 0.000008 |
| Otu00129 | 0.00 | 0.00 | 0.00 | 0.00 | 0.00 | 0.00 | Firmicutes | Phascolarctobacterium | 0.000012 |
| Otu00130 | 0.00 | 0.00 | 0.00 | 0.00 | 0.00 | 0.00 | Proteobacteria | Klebsiella | 0.000008 |
| Otu00132 | 0.00 | 0.00 | 0.00 | 0.00 | 0.00 | 0.00 | Firmicutes | Dialister | 0.000016 |
| Otu00133 | 0.00 | 0.00 | 0.00 | 0.00 | 0.00 | 0.00 | Bacteroidetes | Alistipes | 0.000007 |
| Otu00134 | 0.00 | 0.00 | 0.00 | 0.00 | 0.00 | 0.00 | Firmicutes | Acidaminococcus | 0.000009 |
| Otu00135 | 0.00 | 0.00 | 0.00 | 0.00 | 0.00 | 0.00 | Actinobacteria | Gordonibacter | 0.000009 |
| Otu00138 | 0.00 | 0.00 | 0.00 | 0.00 | 0.00 | 0.00 | Bacteroidetes | Bacteroides | 0.000006 |
| Otu00139 | 0.00 | 0.00 | 0.00 | 0.00 | 0.00 | 0.00 | Proteobacteria | Sutterella | 0.000008 |
| Otu00140 | 0.00 | 0.00 | 0.00 | 0.00 | 0.00 | 0.00 | Firmicutes | unclassified from Lachnospiraceae | 0.000006 |
| Otu00142 | 0.00 | 0.00 | 0.00 | -2.11 | 0.00 | 0.00 | Firmicutes | Veillonella | 0.000008 |
| Otu00143 | 0.00 | 0.00 | 0.00 | 0.00 | 0.00 | 0.00 | Proteobacteria | Sutterella | 0.000009 |
| Otu00144 | 0.00 | 0.00 | 0.00 | 0.00 | 0.00 | 0.00 | Firmicutes | unclassified from Lachnospiraceae | 0.000014 |
| Otu00145 | 0.00 | 0.00 | 0.00 | -2.07 | 0.00 | 0.00 | Firmicutes | Veillonella | 0.000008 |
| Otu00147 | 0.00 | 0.00 | 0.00 | 0.00 | 0.00 | 0.00 | Firmicutes | Acidaminococcus | 0.000005 |
| Otu00148 | 0.00 | 0.00 | 0.00 | 0.00 | 0.00 | 0.00 | unclassified | unclassified | 0.000010 |
| Otu00149 | 0.00 | 0.00 | 0.00 | 0.00 | 0.00 | 0.00 | unclassified | unclassified | 0.000010 |
| Otu00150 | 0.00 | 0.00 | 0.00 | 0.00 | 0.00 | 0.00 | Firmicutes | Flavonifractor | 0.000010 |
| Otu00151 | 0.00 | 0.00 | 0.00 | 0.00 | 0.00 | 0.00 | Actinobacteria | Enterorhabdus | 0.000007 |
| Otu00152 | 0.00 | 0.00 | 0.00 | -2.08 | 0.00 | 0.00 | Proteobacteria | Sutterella | 0.000005 |
| Otu00153 | 0.00 | 0.00 | 0.00 | 0.00 | 0.00 | 0.00 | Bacteroidetes | Bacteroides | 0.000007 |
| Otu00154 | 0.00 | 0.00 | 0.00 | 0.00 | 0.00 | 0.00 | Firmicutes | Peptococcus | 0.000007 |
| Otu00156 | 0.00 | 0.00 | 0.00 | 0.00 | 0.00 | 0.00 | Firmicutes | Lactonifactor | 0.000006 |
| Otu00157 | 0.00 | 0.00 | 0.00 | 0.00 | 0.00 | 0.00 | Firmicutes | Veillonella | 0.000007 |
| Otu00158 | 0.00 | 0.00 | 0.00 | 0.00 | 0.00 | 0.00 | Firmicutes | Erysipelotrichaceae_incertae_sedis | 0.000009 |
| Otu00161 | 0.00 | 0.00 | 0.00 | 0.00 | 0.00 | 0.00 | Firmicutes | Veillonella | 0.000006 |
| Otu00164 | 0.00 | 0.00 | 0.00 | 0.00 | 0.00 | 0.00 | Firmicutes | unclassified from Clostridiales | 0.000004 |
| Otu00169 | 0.00 | 0.00 | 0.00 | 0.00 | 0.00 | 0.00 | Firmicutes | Blautia | 0.000007 |
| Otu00170 | 0.00 | 0.00 | 0.00 | 0.00 | 0.00 | 0.00 | Firmicutes | Veillonella | 0.000007 |
| Otu00173 | 0.00 | 0.00 | 0.00 | 0.00 | 0.00 | 0.00 | Firmicutes | Dialister | 0.000005 |
| Otu00174 | 0.00 | 0.00 | 0.00 | 0.00 | 0.00 | 0.00 | Proteobacteria | Sutterella | 0.000005 |
| Otu00175 | 0.00 | 0.00 | 0.00 | 0.00 | 0.00 | 0.00 | Firmicutes | unclassified from Lactobacillales | 0.000005 |
| Otu00176 | 0.00 | 0.00 | 0.00 | 0.00 | 0.00 | 0.00 | Firmicutes | Veillonella | 0.000005 |
| Otu00177 | 0.00 | 0.00 | 0.00 | 0.00 | 0.00 | 0.00 | Proteobacteria | Sutterella | 0.000005 |
| Otu00186 | 0.00 | 0.00 | 0.00 | 0.00 | 0.00 | 0.00 | Firmicutes | Streptococcus | 0.000005 |
| Otu00199 | 0.00 | 0.00 | 0.00 | 0.00 | 0.00 | 0.00 | Bacteroidetes | Porphyromonas | 0.000005 |
| Otu00200 | 0.00 | 0.00 | 0.00 | 0.00 | 0.00 | 0.00 | Firmicutes | unclassified from Clostridiales | 0.000004 |
| Otu00202 | 0.00 | 0.00 | 0.00 | 0.00 | 0.00 | 0.00 | Firmicutes | Flavonifractor | 0.000005 |
| Otu00204 | 0.00 | 0.00 | 0.00 | 0.00 | 0.00 | 0.00 | Firmicutes | unclassified from Lachnospiraceae | 0.000004 |
| Otu00209 | 0.00 | 0.00 | 0.00 | 0.00 | 0.00 | 0.00 | Firmicutes | Veillonella | 0.000004 |
| Otu00215 | 0.00 | 0.00 | 0.00 | 0.00 | 0.00 | 0.00 | Bacteroidetes | Bacteroides | 0.000004 |
| Otu00223 | 0.00 | 0.00 | 0.00 | 0.00 | 0.00 | 0.00 | Firmicutes | Finegoldia | 0.000005 |
| Otu00232 | 0.00 | 0.00 | 0.00 | 0.00 | 0.00 | 0.00 | Firmicutes | unclassified from Lachnospiraceae | 0.000004 |
| Otu00266 | 0.00 | 0.00 | 0.00 | 0.00 | 0.00 | 0.00 | Firmicutes | Veillonella | 0.000004 |


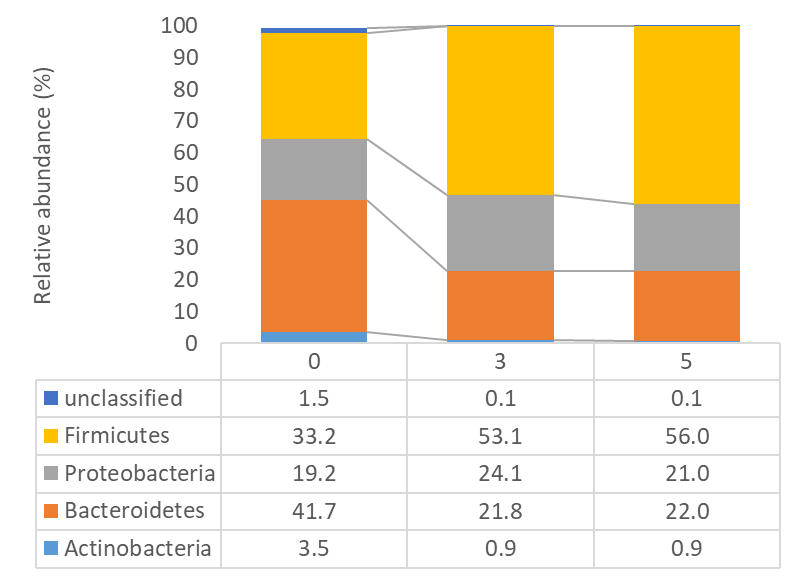


WCAB

ABB


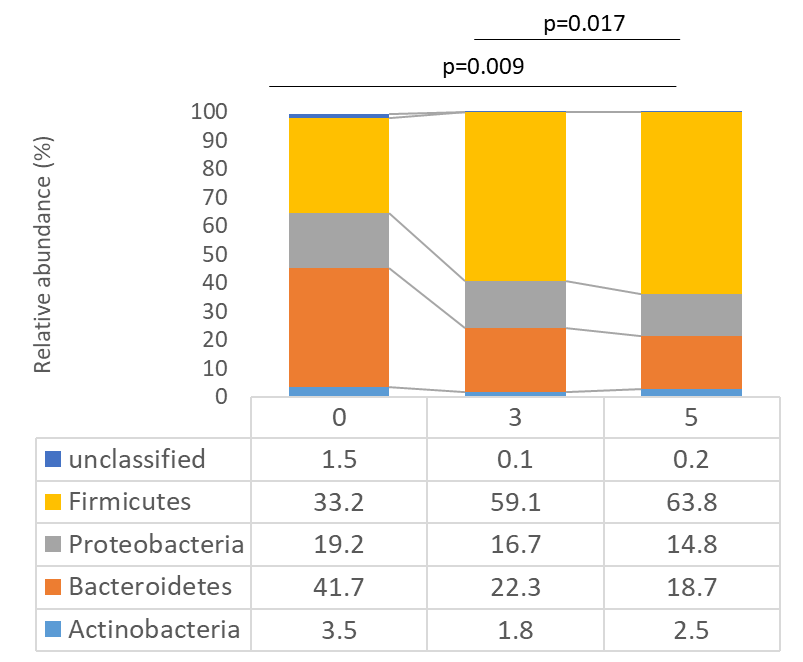


a

**
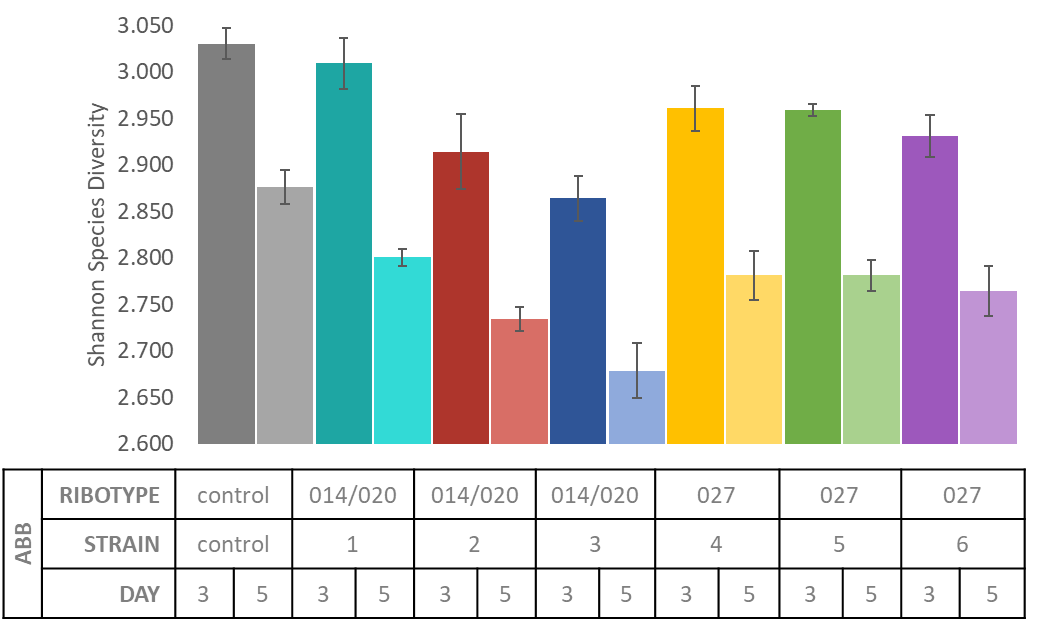

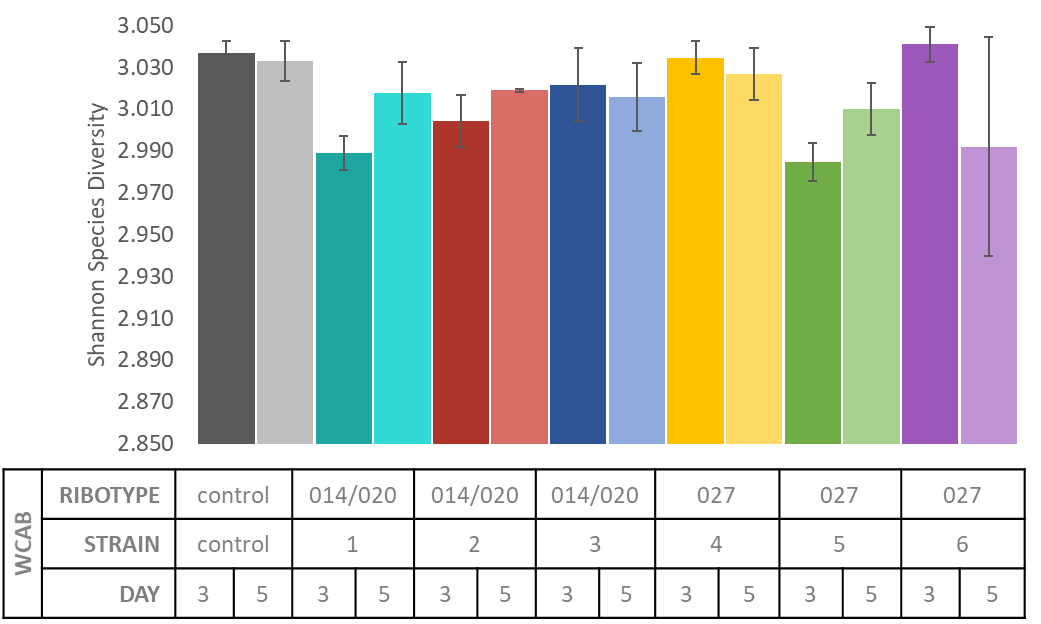
**

b

**Figure S1**: ABB (Anaerobe Basal Broth) growth medium is less suitable than WCAB (Wilkins Chalgren Anaerobe Broth) growth medium for fecal microbiota cultivation. **(a)** The composition of fecal microbiota on phylum level in original pooled faecal sample before any cultivation (point 0) and comparison with microbiota on day 3 and day 5 in WCAB and ABB control medium. All phyla were retained in *in vitro* model but in different abundance. Changes were significant in ABB growth medium, however, there were no statistical differences in composition between samples when using WCAB growth medium (AMOVA: p > 0.05). **(b)** Species diversity (Shannon diversity index) in control medium in comparison to conditioned medium of six different *C. difficile* strains (1-6) belonging to two different ribotypes (014/020, 027) after 3 and 5 days of incubation. In ABB medium the diversity was considerably decreased already in unconditioned control medium and this is very likely associated with substantial further decrease of diversity in conditioned media.

| Comparison 1: Control vs R014 / 020 vs R027 |
| --- |

Classification rules (WEKA jRip):

IF (*Escherichia_Shigella* (Otu00001) >= 0.16428) THEN Ribotype=Control

ELSE IF (*Phascolarctobacterium* (Otu00006) >= 0.047796) THEN Ribotype=R_027

ELSE IF (*Clostridium_sensu_stricto* (Otu00018) <= 0.001429) THEN Ribotype=R_027

ELSE Ribotype=R_014/020

Ranking of OTUs (WEKA ReliefF):

| **Number** | **Average merit** | **Average rank** | **Attribute** | **Taxonomy** |
| --- | --- | --- | --- | --- |
| 1 | 0.258 +- 0.018 | 1.3 +- 0.46 | Otu00018 | *Clostridium_sensu_stricto* |
| 2 | 0.252 +- 0.018 | 1.9 +- 0.54 | Otu00025 | *Bacteroides* |
| 3 | 0.231 +- 0.017 | 3.8 +- 1.17 | Otu00001 | *Escherichia_Shigella* |
| 4 | 0.223 +- 0.014 | 4.7 +- 0.64 | Otu00027 | *Coprococcus* |
| 5 | 0.218 +- 0.017 | 5.7 +- 1.27 | Otu00005 | *Bacteroides* |
| 6 | 0.216 +- 0.02 | 6 +- 1.26 | Otu00002 | *Bacteroides* |
| 7 | 0.207 +- 0.024 | 6.7 +- 2.49 | Otu00096 | *Streptococcus* |
| 8 | 0.188 +- 0.016 | 10.3 +- 2 | Otu00059 | *Lactobacillus* |
| 9 | 0.188 +- 0.022 | 10.7 +- 4.34 | Otu00083 | *Erysipelotrichaceae_incertae_sedis* |
| 10 | 0.184 +- 0.011 | 10.9 +- 1.87 | Otu00020 | *Flavonifractor* |

| Comparison 2: Control vs R014 / 020 |
| --- |

Classification rules (WEKA jRip):

IF (*Escherichia_Shigella* (Otu00001) >= 0.16428) THEN Ribotype=Control

ELSE Ribotype=R_014/020

Ranking of OTUs (WEKA ReliefF):

| **Number** | **Average merit** | **Average rank** | **Attribute** | **Taxonomy** |
| --- | --- | --- | --- | --- |
| 1 | 0.741 +- 0.02 | 1.3 +- 0.46 | Otu00018 | *Clostridium_sensu_stricto* |
| 2 | 0.717 +- 0.006 | 2 +- 0.63 | Otu00025 | *Bacteroides* |
| 3 | 0.663 +- 0.024 | 3.6 +- 0.66 | Otu00001 | *Escherichia_Shigella* |
| 4 | 0.629 +- 0.02 | 5.2 +- 1.33 | Otu00005 | *Bacteroides* |
| 5 | 0.616 +- 0.022 | 5.7 +- 1.49 | Otu00002 | *Bacteroides* |
| 6 | 0.619 +- 0.026 | 5.9 +- 1.7 | Otu00045 | *uncl. from Burkholderiales* |
| 7 | 0.607 +- 0.073 | 7.3 +- 2.53 | Otu00096 | *Streptococcus* |
| 8 | 0.577 +- 0.015 | 8.2 +- 1.33 | Otu00012 | *Enterococcus* |
| 9 | 0.583 +- 0.064 | 9.3 +- 3.1 | Otu00083 | *Erysipelotrichaceae_incertae_sedis* |
| 10 | 0.56 +- 0.011 | 10.4 +- 1.69 | Otu00027 | *Coprococcus* |

| Comparison 3: Control vs R027 |
| --- |

Classification rules (WEKA jRip):

IF (*Escherichia_Shigella* (Otu00001) >= 0.16428) THEN Ribotype=Control

ELSE Ribotype=R_027

Ranking of OTUs (WEKA ReliefF):

| **Number** | **Average merit** | **Average rank** | **Attribute** | **Taxonomy** |
| --- | --- | --- | --- | --- |
| 1 | 0.752 +- 0.02 | 1.1 +- 0.3 | Otu00018 | *Clostridium_sensu_stricto* |
| 2 | 0.704 +- 0.022 | 2.7 +- 0.9 | Otu00001 | *Escherichia_Shigella* |
| 3 | 0.695 +- 0.007 | 3 +- 0.89 | Otu00025 | *Bacteroides* |
| 4 | 0.624 +- 0.029 | 4.7 +- 1.19 | Otu00005 | *Bacteroides* |
| 5 | 0.622 +- 0.071 | 4.9 +- 1.7 | Otu00096 | *Streptococcus* |
| 6 | 0.599 +- 0.041 | 6 +- 1.26 | Otu00002 | *Bacteroides* |
| 7 | 0.571 +- 0.036 | 7.2 +- 1.33 | Otu00059 | *Lactobacillus* |
| 8 | 0.549 +- 0.025 | 8.9 +- 1.04 | Otu00065 | *uncl. From Ruminococcaceae* |
| 9 | 0.556 +- 0.061 | 9.2 +- 2.86 | Otu00089 | *Odoribacter* |
| 10 | 0.538 +- 0.015 | 9.8 +- 1.78 | Otu00019 | *Streptococcus* |

**Figure S2**: The WEKA jRip approach was used to analyse what OTUs are important for determining ribotype in three dataset variants (Comparisons 1, 2 and 3). Comparison 1 considers all three types of samples, Comparison 2 ribotype 014/020 vs. controls and Comparison 3 ribotype 027 vs controls. The jRip method selects a very short list of OTUs by learning classification rules (also called IF/THEN rules). Rules are used to make predictions as well as determine differentiating attributes (OTUs), w.r.t. the ribotype. Rules inside the rule set should be used sequentially, e.g. in Comparison 1, the rules state that if relative abundance of *Escherichia_Shigella* is greater than 0.16428, that particular example is predicted as control. If not, continue to the next rule which states that if *Phascolarctobacterium* abundance is greater than 0.47796, or *Clostridium sensu stricto* is lower than 0.001429, predict ribotype 027. If even that does not hold, predict ribotype 014/020. In addition, the WEKA ReliefF approach was used to rank the OTUs in terms of their importance for distinguishing among/between the considered types of samples in the respective comparisons: top 10 ranked OTUs are displayed for each comparison.The three tables with rankings of OTUs contain rows which are displayed in red. Those rows represent OTUs that are present in classification rules and have been determined as important for differentiation between controls and ribotypes. The jRip and ReliefF algorithms are explained in detail below in section Machine learning methods. The section on jRip also includes descriptions of input datasets (Comparisons).

**
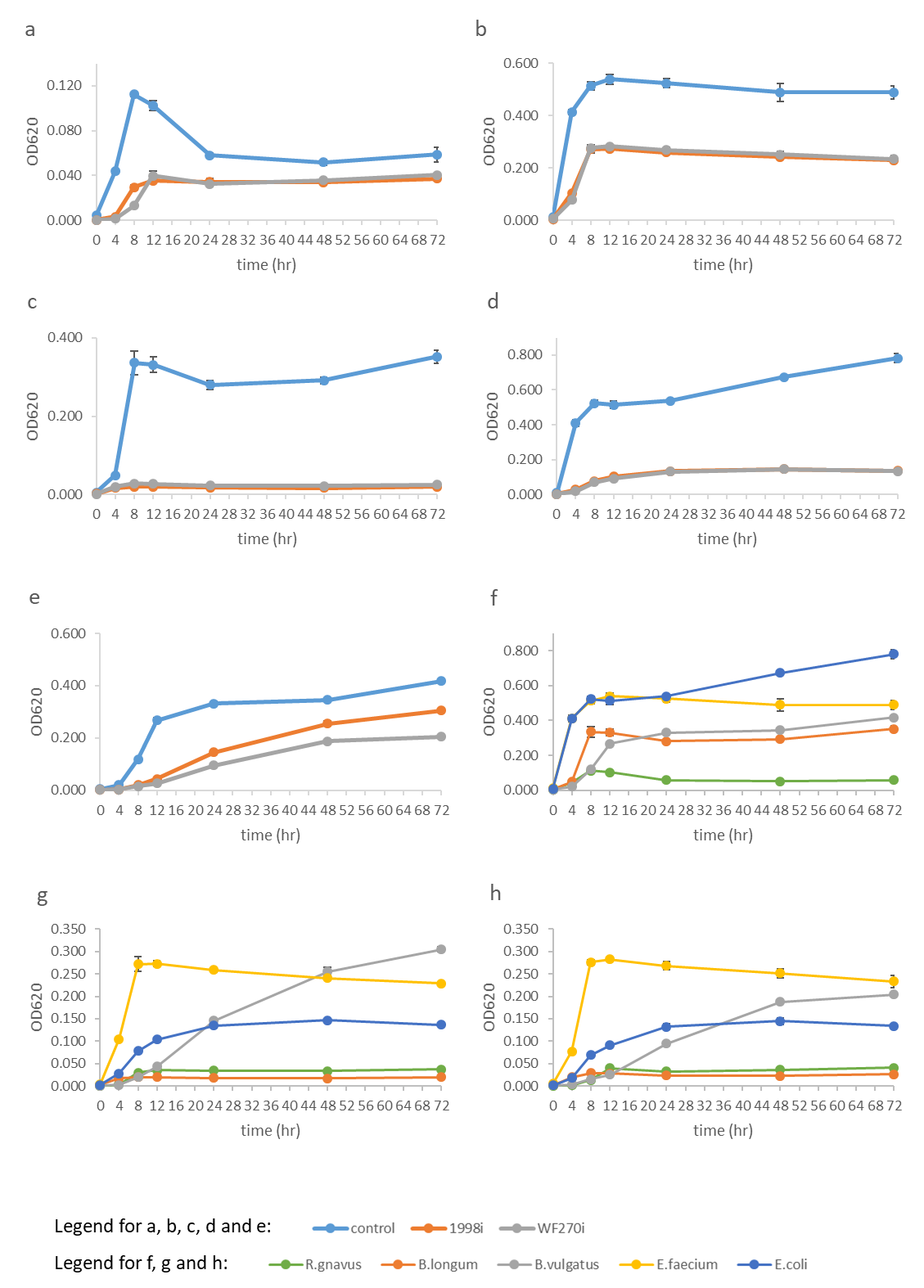
**

**Figure S3**: *In vitro* growth of commercial *Ruminococcus gnavus* (a) and four different isolates from fecal material (*Enterococcus faecium*, b; *Bifidobacterium longum*, c; *E. coli*, d; *Bacteroides vulgatus*, e) in Wilkins Chalgren Anaerobe Broth (WCAB) control medium (blue line), WCAB conditioned medium of *C. difficile* ribotype 027 (strain 1998, orange line) and in WCAB conditioned medium of *C. difficile* ribotype 014/020 (strain WF270, grey line). Panels f, g and h represent *in vitro* growth of *R. gnavus, B. longum, B. vulgatus, E. faecium* and *E. coli* in WCAB control medium (f), WCAB conditioned medium of *C. difficile* ribotype 027 (g) and in WCAB conditioned medium of *C. difficile* ribotype 014/020 (h), respectively. Error bars represent the mean ± standard deviation of triplicate experiments.

Legend for f, g and h:


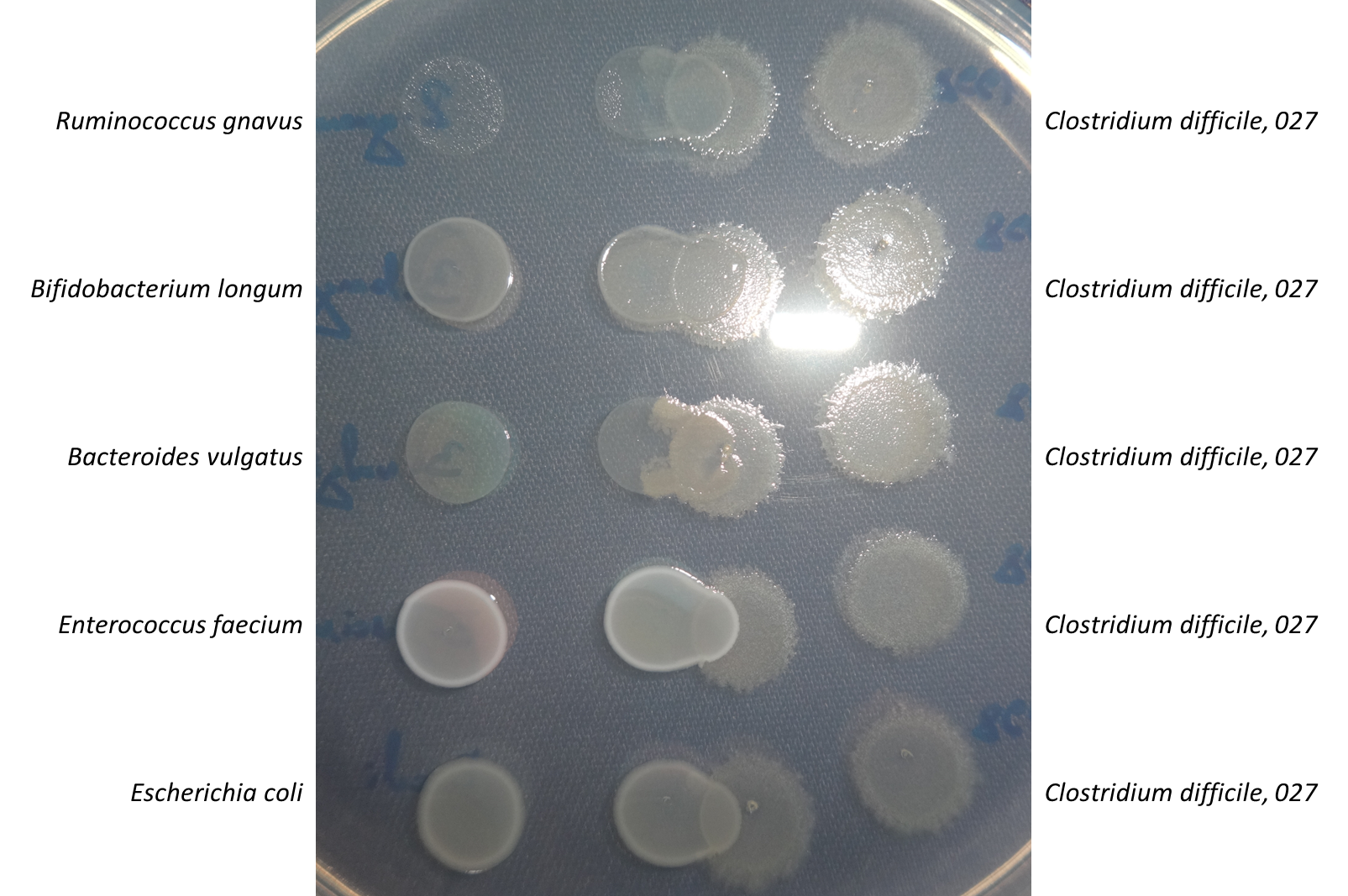


**Figure S4**: Co-cultures of *C. difficile* ribotype 027 with four different isolates from fecal material (*Bifidobacterium longum*, *Bacteroides vulgatus*, *Enterococcus faecium*, *E. coli*) and *Ruminococcus gnavus* on Wilkins Chalgren Anaerobe Agar (WCAA) plate. No direct inhibition is observed except for *R. gnavus*.

**
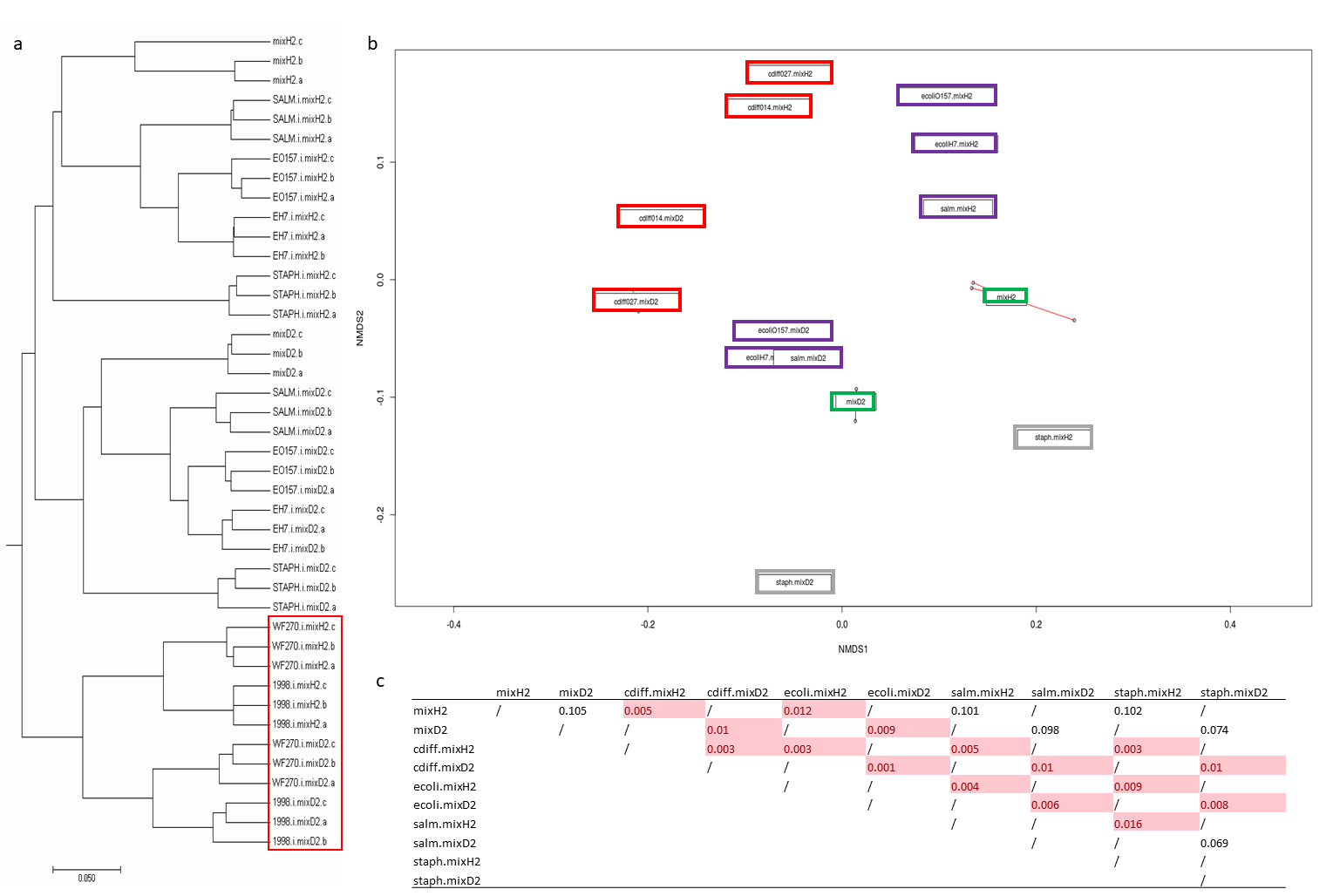
**

**Figure S5:** (a) Hierarchical clustering of samples before antibiotic therapy (mixH2) and after antibiotic therapy (mixD2) cultured in WCAB (Wilikins Chalgren Anaerobe Broth) conditioned media of four different bacteria (*Clostridium difficile*; *E. coli*; *Salmonella* Enteritidis; *Staphylococcus epidermidis*), obtained by MEGA software. Red square indicate samples of microbiota mixH2 and mixD2 grown in WCAB conditioned media of two *C. difficile* ribotypes (027, strain 1998 and 014/020, strain WF270). (b) Non-Metric Multidimensional Scaling (NMDS) analysis (R, vegan package) for microbiota samples before antibiotic therapy (mixH2) and after antibiotic therapy (mixD2) cultured in WCAB conditioned media of four different bacteria (*C. difficile*, cdiff, red squares; *E. coli*, ecoli, purple squares; *S.* Enteritidis, salm, purple squares; *S. epidermidis*, staph, grey squares). Green squares indicate control samples. (c) AMOVA test (mothur software) for samples of microbiota mixH2 and mixD2 cultured in WCAB conditioned media of four different bacteria (*C. difficile*, cdiff; *E. coli*, ecoli; *S.* Enteritidis, salm; *S. epidermidis*, staph). Significant differences between samples (p < 0.05) are marked with red color.

**Machine learning methods**

Below we provide brief descriptions of the three machine learning approaches used in this study. We applied three types of machine learning methods to analyze the collected data. In machine learning terminology, each data sample is called an example and corresponds to a row in the data table. The variables, also called attributes, correspond to columns in the data table. We distinguish between descriptive variables (inputs) and target variables (outputs). In this study, we used incubation period and type of sample (control, ribotype 014/020 and ribotype 027) as descriptive variables and the bacterial community composition in terms of the relative abundance of OTUs as target variables.

**Predictive Clustering Trees (PCT).^1^** PCTs are multi-target decision trees implemented in the CLUS software, where a tree is viewed as a hierarchy of clusters (nodes). A PCT is capable of predicting multiple values simultaneously (one for each target attribute). The root node represents one large cluster of all data which is recursively split into smaller clusters. The tree is partitioned by calculating the heuristic value of every possible test (split) point and selecting the one with the best heuristic value. The partitioning continues until a stopping criterion is met. Nodes (clusters) at the bottom are called leaf nodes and provide predictions, which are the mean/mode values of each target attribute in the cluster. The heuristic that guides the test (split) selection is to minimize intra-cluster variation summed over the subsets induced by the test. Lower average variation in the resulting clusters yields more accurate models.

A general overview of the PCT induction algorithm is shown below. The dataset is input to the PCT procedure which starts tree construction by searching for the best possible test to split the data (line 1 of PCT procedure). If a test is found, the data are split into subsets according to the conditions in the test (i.e., *variableX > valueA*). The algorithm recursively builds the tree by calling itself on these subsets (line 4 of the PCT procedure). It proceeds in this fashion until the stopping criterion is met. The best possible test is selected by evaluating all input features and all possible split points for them. The selected test is the one that reduces the variance (i.e., impurity) the most. The variance reduction caused by the candidate test (i.e., test that is being evaluated) is calculated by splitting the data according to it and calculating the variances of all branches (resulting from the splitting). The calculated variances of all branches are then summed and subtracted from the variance calculated before splitting with the candidate test (line 4 of BestTest procedure). If the evaluation shows that the new split yields better reduction of variance, it is remembered (line 6 of BestTest procedure) and discarded otherwise. The result of this algorithm is a predictive clustering tree: the tree built from our data is shown in the paper in Figure 2a.

The input features used by the PCT induction algorithm were the incubation period and the ribotype. We used them to predict the relative abundances of the OTUs. An example of the input data for the PCT induction algorithm is shown below.

| INPUT FEATURES | | OUTPUT FEATURES | | | | | |
| --- | --- | --- | --- | --- | --- | --- | --- |
| Incubation period | Ribotype | OTU1 | OTU2 | OTU3 | OTU4 | … | OTU161 |
| 3 | Control | 0.093041 | 0.167374 | 0.119619 | 0.075336 | … | 0 |
| 3 | 027 | 0.089895 | 0.163262 | 0.12125 | 0.066301 | … | 0 |
| 3 | 041/020 | 0.097072 | 0.163702 | 0.113893 | 0.07319 | … | 0 |
| 5 | Control | 0.079509 | 0.16778 | 0.133853 | 0.062102 | … | 0 |
| 5 | 027 | 0.081908 | 0.159606 | 0.115814 | 0.053138 | … | 0.000039 |

**RIPPER (WEKA implementation).** Classification rules are if/then statements, where the antecedent part contains some conditions on the attributes (descriptive variables) and the consequent part predicts a value for the class (target variable). A rule distinguishes examples of the predicted class from those of other classes. An example rule is the following: IF (Otu00022 >= 0.011133) THEN Ribotype=R_027 (which predicts that the example is of type ribotype 027 if the OTU score, i.e. relative abundance, is at least 0.011133). Classification rule algorithms construct sets of such rules. Each rule applies to (explains) several examples. The rules can be constructed directly from the training examples in an iterative process which constructs one rule at a time, removes the examples explained by the rule and repeats the process until all examples are explained. We used the jRip (WEKA RIPPER algorithm implementation)^2^ for learning rule sets. We set the minimum number of examples to be explained (covered) by a rule to 2 and the number of optimization runs to 2. For the purpose of learning classification rules, we used the relative abundances of OTUs as input features and ribotype as the output (target) variable. We learned classification rules with three variants of input data (Comparisons), where we considered different combinations of ribotype and control examples. In Comparison 1, classification rules were constructed using all examples. An excerpt of the input data for Comparison 1 is shown below.

| INPUT FEATURES | | | | | | TARGET |
| --- | --- | --- | --- | --- | --- | --- |
| OTU1 | OTU2 | OTU3 | OTU4 | … | OTU161 | Ribotype |
| 0.093041 | 0.167374 | 0.119619 | 0.075336 | … | 0 | Control |
| 0.089895 | 0.163262 | 0.12125 | 0.066301 | … | 0 | 027 |
| 0.097072 | 0.163702 | 0.113893 | 0.07319 | … | 0 | 014/020 |
| 0.079509 | 0.16778 | 0.133853 | 0.062102 | … | 0 | Control |
| 0.081908 | 0.159606 | 0.115814 | 0.053138 | … | 0.000039 | 027 |

In Comparison 2 and 3, we compare control samples to samples with ribotype 014/020 and 027, respectively. The datasets for Comparison 2 and 3 are created by taking all data and omitting samples with ribotype 027 and ribotype 014/020 respectively.

The RIPPER algorithm (as implemented in Weka) works in the following way. First, it initializes an empty rule set. After that, the algorithm has two stages: building stage and optimization stage. In the building stage, the rules are first grown and then immediately pruned. This stage continues until there are no more positive examples or if the error rate or the rule being added is greater than 50% or if the description length of the ruleset is 64 bits greater than the smallest description length seen so far. The input data is split into four parts: positive (1) and negative (2) examples for the growing phase and positive (3) and negative (4) examples for the pruning phase. When growing a rule, the algorithm greedily adds tests (i.e., conditions) to the rule until the rule has 100% accuracy. Then, the algorithm removes unneccessary conditions from the rule by testing against the pruning data sets. In case of more than two classes (which is Comparison 1 in our case), the classess are ordered by the increasing number of examples belonging to them. The algorithm then learns a rule set for the least prevalent class by trying to separate examples from all the other classess. The examples covered by the rule set for the aforementioned class are removed and the procedure continues by learning a rule set for the second least prevalent class, tries to separate examples of this class from all remaining classess. When only one class remains, it is proclaimed as the default class. In the optimization stage, two variants of each rule in the rule set are generated and pruned: one that adds conditions to the rule itself and second one that adds conditions to an empty rule. Both variants are grown as described earlier. The variant with the smallest description length is selected as the final rule in the ruleset. After examinaton of all the rules, it is possible that positive examples are left uncovered. In that case, more rules are generated as described before.

**ReliefF (WEKA implementation).** The WEKA package also includes different approaches for estimating the relevance of attributes for a classification task at hand. One such approach is the Recursive Elimination of Features-F (ReliefF)^3^ which calculates for each attribute a merit value estimating the relevance of the attribute for distinguishing between the selected target groups (class values). This merit value takes into account the interaction of the attribute with other attributes. By sorting ReliefF merits from highest to lowest we obtain a ranked list of attributes. A ranking is constructed based on average merit/rank across ten subsets of the dataset, each comprising 90% of the samples and corresponding to one of the ten training dataset in 10-fold cross-validation. The input data for the feature ranking was the same as for Comparison 1: All OTUs and ribotypes.

The ReliefF algorithm belongs to the Relief family of algorithms. The variant we used (ReliefF) improves the initial version by being able to handle multiclass problems as well as adding capability to handle incomplete and noisy data. The ReliefF algorithm estimates the quality of attributes according to how well they distinguish between instances that are near to each other. The algorithm searches for k nearest neighbors (called hits H_j_, line 4 of Algorithm 3) of the same class as the randomly selected instance R_i_ (line 3) and k nearest neighbors for each of the other classess (called nearest missess M_j_(C) on lines 5-6 of Algorithm 3). It then updates the quality estimation W[A] of all attributes based on the values of hits, misses and randomly selected instance R_i_ (lines 8-11). The reasoning behind this is the following: If instances R_i_ and H_j_ have different values of the examined attribute, then this attribute separates instances of the same class. Such behavior is not desired, so we decrease the quality of that attribute. On the other hand, when R_i_ and M_j_ have different values for the examined attribute, the attribute is rewarded. The whole process is repeated m-times, where m is a user-defined parameter. In our setup, we used m=’number of examples in the dataset’. The output of the algorithm is a vector that estimates the quality of all attributes. Table 1 in the paper contains top 20 ranked attributes, i.e., OTUs, in terms of their relevance for distinguishing between control, ribotype 027, and ribotype 014/020 samples (Comparison 1).

1. Blockeel, H. & De Raedt, L. Top-down induction of first-order logical decision trees. *Artificial Intelligence* **101**, 285-297, doi:10.1016/S0004-3702(98)00034-4 (1998).
2. Cohen, W. W. Fast effective rule induction, in *Proceedings of the 12th International Conference on Machine Learning.* (eds Prieditis A & Russell S) 115-123 (ACM Press New York).
3. Robnik-Sikonja, M. & Kononenko, I. Theoretical and empirical analysis of ReliefF and RReliefF. *Machine Learning* **53**, 23-69, doi:10.1023/A:1025667309714 (2003).
